# The investigation of human cerebrospinal fluid exosome in spinal cord injury

**DOI:** 10.1172/jci.insight.205134

**Published:** 2026-06-23

**Authors:** Dallas L. Sheinberg, Haichao Wei, Joseph S. Withrow, Farshad Homayouni Moghadam, Chia-Chen Lu, Jyotirmoy Rakshit, Jennifer Zaragoza, John R. Williams, Wen Li, Jacques J. Morcos, Jia Qian Wu

**Affiliations:** 1 Department of Neurosurgery, McGovern Medical School, and; 2 Center for Neuroimmunology and Glial Biology, Institute of Molecular Medicine, University of Texas Health Science Center, Houston, Texas, USA.; 3 Carolina Neurosurgery and Spine Associates, Charlotte, North Carolina, USA.; 4 Division of Clinical and Translational Sciences, Department of Internal Medicine, University of Texas McGovern Medical School, Houston, Texas, USA.; 5 MD Anderson Cancer Center UTHealth Graduate School of Biomedical Sciences, Houston, Texas, USA.

**Keywords:** Cell biology, Neuroscience, Bioinformatics, Biomarkers

## Abstract

Spinal cord injury (SCI) leads to severe neurological and functional impairments, yet reliable biomarkers for assessing injury severity and predicting recovery remain limited. Cerebrospinal fluid (CSF) is in direct contact with the central nervous system and provides a valuable source for detecting molecular changes after SCI. Although exosomal microRNAs (miRNAs) and proteins are increasingly recognized as mediators of intercellular communication, the role of human CSF exosomes in SCI has not been systematically investigated. To identify exosome-based biomarkers and potential therapeutic targets, we analyzed CSF and serum exosomes from patients with acute SCI using RNA sequencing and proteomic profiling. Weighted gene co-expression network analysis identified 6 gene modules significantly associated with injury severity and neurological recovery at 3 months. Proteomic analysis revealed a 5-protein panel that distinguished complete from incomplete SCI and a 4-protein panel that predicted neurological improvement. Additionally, 15 CSF-specific and 9 serum-specific exosomal miRNAs were identified independent of injury severity. Among 10 tested miRNAs associated with neurological recovery, 7 regulated astrocyte proliferation, and 6 promoted neurite extension and synapse formation. Overall, this study provides a comprehensive characterization of CSF exosomal miRNAs and proteins in human SCI and identifies molecular signatures associated with injury severity and recovery.

## Abstract 2

Researchers identified molecules in cerebrospinal fluid that help predict spinal cord injury severity and recovery, opening new possibilities for diagnosis and treatment.

## Introduction

Traumatic spinal cord injury (SCI) occurs in 12,000 people per year in the United States, with profound long-term physical, psychological, and economic burdens on the patient and the health care system (1, 2). Estimates of the lifetime cost to care for someone with an SCI range from $325,000 to $1.35 million, and the yearly cost to society reaches $8 billion (1, 3, 4). Following the initial traumatic injury, a complex cascade of secondary injury occurs involving vascular damage and ischemia, excitotoxicity, neural inflammation, demyelination, and glial scar formation (5).

Despite advances in understanding SCI, randomized controlled trials of therapeutic interventions have proven to be challenging as a result of injury heterogeneity and reliance on functional neurological examination (6, 7). Early assessment of impairment following SCI is standardized with MRI and neurological examination using the American Spinal Injury Association (ASIA) exam (8). The gold standard in predicting long-term functional deficits is the ASIA Impairment Scale (AIS) grade, which has limitations when applied to clinical trials owing to multiple variables in the acute phase, such as comorbid injuries and patient unresponsiveness.

Because of the suboptimal reliability of current methods of early evaluation, blood and cerebrospinal fluid (CSF) biomarkers that classify injury severity and predict recovery are actively being investigated. Exosomes are small membrane-derived extracellular vesicles (EVs) that participate in cell-to-cell transfer of biomolecules such as protein and RNA, serving as critical mediators after SCI. Exosomes have emerged as promising therapeutics in various diseases. Studies have shown that exosomes facilitate pathways including apoptosis, angiogenesis, microglial migration, and astrogliosis, among others (9). The role of exosomes in these pathological processes involves the transfer of RNAs and proteins between cells in the central nervous system (CNS), protecting them from RNase degradation (10). Exosomal RNAs are known to be more stable than free RNA, resulting in lasting effects on disease-related alterations in gene expression (11).

MicroRNAs (miRNAs) are small non-coding RNAs that regulate gene expression and protein synthesis. Changes in miRNA expression play a key role in injury (12) due to miRNA regulation of numerous physiological functions. miRNAs have been studied in a wide range of disease processes as biomarkers for diagnosis and prognosis owing to their stability in human fluids and tissue specificity (13, 14). In SCI, the majority of studies are limited to animal models and use blood for profiling. Tigchelaar et al. described miRNA expression changes in serum and CSF in humans after SCI, demonstrating biomarkers for injury severity and neurological outcome prognosis (13). Currently, there are no publications on CSF exosomal miRNA and proteins after SCI.

In the present study, we investigated the profile of exosomes in CSF and serum after acute SCI using a combination of cellular and molecular techniques. A comprehensive analysis of the exosome transcriptome was conducted at different time points during the acute phase of SCI to identify modulator pathways and pathways that may promote secondary injury. Furthermore, we evaluated CSF exosome protein expression patterns across injury severities and time to provide a comprehensive understanding of how expression evolves in the acute phase of injury and its relationship to functional outcomes at 3 months follow-up. The roles of many of the miRNAs identified in this study remain poorly characterized, with limited prior research available regarding their impact on neuroregeneration. Astrocytes are known to be a major cell type in the spinal cord interacting with neurons (15, 16). Following SCI, astrocytes rapidly become reactive, undergo significant changes, and influence neuronal recovery (15, 16). Employing assays focusing on astrocyte activation and astrocyte-neuron interactions, we demonstrated 7 miRNAs differentially expressed in improved patients that regulated astrocyte proliferation and 6 that exhibited dual roles in promoting neurite extension and synapse formation. Altogether, this study investigated CSF exosomal miRNAs and proteins in SCI patients, provided valuable insight into SCI pathophysiology, and demonstrated their utility as biomarkers for diagnosis and prognosis, and how SCI could lead to other diseases, such as neurodegenerative diseases.

## Results

### Clinical and demographic characteristics of participants.

The early phase of SCI, spanning the first few days after injury, is a critical window for therapeutic intervention. Biomarkers identified during this phase can aid in assessing injury severity and predicting recovery. CSF and serum are promising sources for such biomarkers. To investigate exosomal miRNA and protein signatures, we collected samples from SCI patients at the Memorial Hermann Emergency Department (Houston, Texas, USA).

A total of 25 traumatic SCI patients were recruited, and their clinical and demographic characteristics are summarized in Supplemental Table 1 (supplemental material available online with this article; https://doi.org/10.1172/jci.insight.205134DS1). Of the 25 patients, 22 (88%) were male and 3 (12%) were female, and the average age was 49.2 ± 19.2 years (mean ± SD). Of all injuries, 21 were cervical, 3 were thoracic, and 1 was lumbar. Baseline neurological assessment showed complete SCI (AIS A) in 44% of patients. In patients with incomplete SCI, 28% were AIS B and 28% AIS C. Detailed neurological assessments were obtained at follow-up to evaluate patient improvement (Supplemental Table 1). The average change in upper extremity, lower extremity, and total motor scores was 8.92 (SD = 15.48), 10.68 (SD = 11.46), and 20.00 (SD = 21.95), respectively. For all included patients, 64% had improvement ≥1 AIS grade at follow-up. In the subgroup of patients with AIS A injury, improvement was seen in 36%.

### Characterization of exosome and comparison of enriched miRNAs in CSF and serum.

Exosomes were extracted from the CSF and serum of SCI patients at 1, 3, and 5 days after injury. Transmission electron microscopy and nanoparticle tracking analysis, both standard techniques in the EV field, were used to assess the structural integrity and size distribution of the isolated particles. The results revealed that the majority of the particles had the structural integrity of the exosomes (Supplemental Figure 1A) and a diameter between 50 and 150 nm (Supplemental Figure 1B). These results indicate that the isolated exosomes were structurally preserved and fell within the expected size range, consistent with the known characteristics of exosomes.

**Figure 1:**
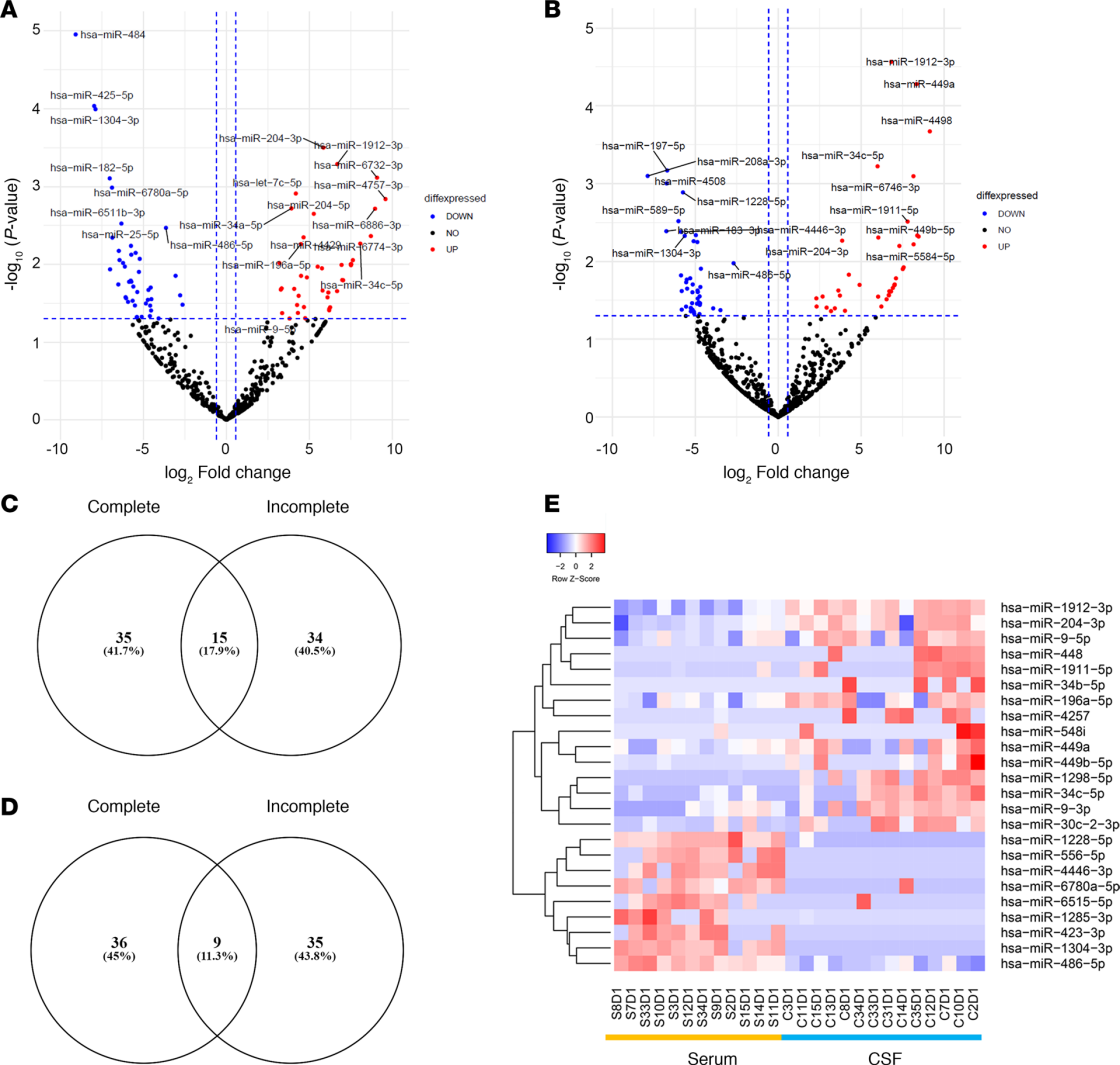
Differential gene expression analysis between serum and CSF. (**A** and **B**) Volcano plots for identified DEGs between CSF and serum in complete injury (**A**) and incomplete injury (**B**). Blue dots represent read counts with fold changes ≤ –1.5 and *P* value < 0.05. Red dots show read counts with fold changes ≥ 1.5 and *P* value < 0.05. (**C** and **D**) The Venn diagram displays the common upregulated DEGs in CSF (**C**) and serum (**D**). (**E**) Heatmap shows 15 genes significantly enriched in CSF and 9 genes in serum.

CSF is in direct contact with the brain and spinal cord, making CSF-derived exosomes particularly enriched in molecules that reflect CNS physiology and pathology (17). In contrast, serum-derived exosomes originate from a broader range of tissues, making it more challenging to attribute their content specifically to CNS-related processes (18). To explore these differences in the context of SCI, we performed a comparative analysis of CSF and serum exosome-derived miRNA expression profiles based on injury severity (complete AIS A vs. incomplete AIS B/C SCI) 1 day after injury (Figure 1, A and B). In the complete injury group, 50 genes were enriched in CSF and 45 in serum. In the incomplete injury group, 49 genes were enriched in CSF and 44 in serum. There were 15 common genes between the complete and incomplete injury conditions (Figure 1, C and E) specifically enriched in CSF samples compared with serum, while there were 9 common genes between the complete and incomplete injury conditions specifically enriched in serum samples (Figure 1, D and E) compared with CSF samples. These findings highlight significant differences in miRNA expression profiles between CSF and serum.

### Proteins in CSF as biomarkers for SCI.

In this study, we used a multiplex immunoassay to measure the abundance of 18 neuronal proteins and 30 cytokines in CSF exosomes (see Methods for details). To assess the clinical utility of these markers, we developed predictive models using receiver operating characteristic (ROC) curve analysis. ROC analysis was conducted to evaluate the discriminatory power of the protein biomarkers in distinguishing between injury severity and recovery outcomes. An area under the curve (AUC) value approaching 1 indicates high sensitivity and specificity, underscoring the potential of these biomarkers for early diagnosis and ongoing monitoring of SCI progression. First, we identified signature proteins associated with injury severity (complete AIS A vs. incomplete AIS B/C SCI) and functional outcome (improvement vs. non-improvement) by performing ROC analysis on all detected proteins and cytokines. Based on these findings, we constructed 2 distinct predictive models using combined protein profiles for early diagnosis and monitoring SCI recovery.

**Figure 2:**
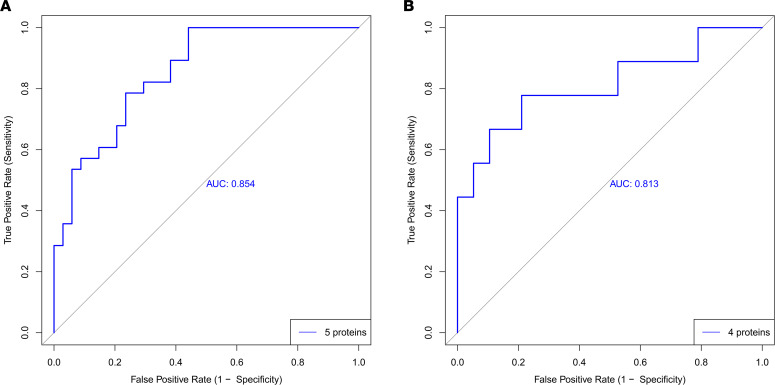
Proteomic profiling of CSF exosomes after SCI. (**A** and **B**) ROC curves illustrate the performance of combination proteins in distinguishing patient outcomes: 5 proteins (amyloid-β_1–42_ + KLK6 + NCAM-1 + IFN-α + IP-10) for differentiating between complete and incomplete injuries (AUC = 0.854) (**A**) and 4 proteins (MIF + HGF + IL-1RA + IL-10) for distinguishing between improvement and non-improvement in the severe group (AUC = 0.813) (**B**).

A prediction model incorporating 5 proteins — amyloid-β_1–42_, KLK6, NCAM-1, IP-10, and IFN-α — demonstrated strong performance in distinguishing complete (AIS A) from incomplete (AIS B/C) patients, achieving an AUC of 0.854 (Figure 2A). These biomarkers reflect a broad spectrum of biological functions, including roles as inflammatory mediators, structural and cell adhesion molecules, and growth factors. For instance, among them, NCAM-1 is a member of the immunoglobulin superfamily and is predominantly expressed in neurons and glial cells (19). It plays critical roles in CNS development, synaptic plasticity, and cell-cell communication (19, 20). Streijger et al. reported significantly decreased NCAM-1 levels in the whole CSF of patients with complete injuries compared with those with incomplete injuries, suggesting a loss or dysfunction of NCAM-1–expressing cells in more severe injury states (21). Notably, the elevations of amyloid-β (Aβ) in CSF exosome can be observed in a very early stage of SCI, which may pave the way for the development of neurodegeneration at a later time. In addition, a separate prediction model using 4 different proteins — MIF, HGF, IL-1RA, and IL-10 — was developed to assess neurological improvement, defined as an AIS grade improvement of greater than 1 at ≥3 months after injury. This model achieved an AUC of 0.813 (Figure 2B). Patients who exhibited neurological recovery showed elevated levels of antiinflammatory and neuroprotective cytokines, particularly IL-1RA and IL-10. These cytokines are known to suppress proinflammatory mediator production, regulate immune cell activation, and support tissue repair and regeneration (22, 23). In preclinical SCI models, administration of IL-1RA or IL-10 has led to reduced lesion volumes and improved motor outcomes, further underscoring their therapeutic potential (22). Together, these biomarker panels offer predictive insight into injury severity and recovery potential, and also highlight molecular pathways that may be targeted to improve outcomes following SCI.

### Construction of weighted co-expression networks and identification of modules associated with injury severity and recovery.

CSF-derived exosomal miRNAs are more likely to reflect CNS-specific processes, making them valuable candidates for biomarkers to assess SCI severity and predict recovery outcomes. In order to investigate exosome miRNAs associated with injury severity, we first addressed potential serum contamination by normalizing all miRNA reads using the transcripts per million method and performing sample clustering to distinguish CSF from serum profiles. Some samples were excluded as a result of contamination with blood. Samples with low read counts (<5) and poor clustering performance were excluded. After quality control, 38 high-quality CSF samples were selected for downstream analysis.

**Figure 3:**
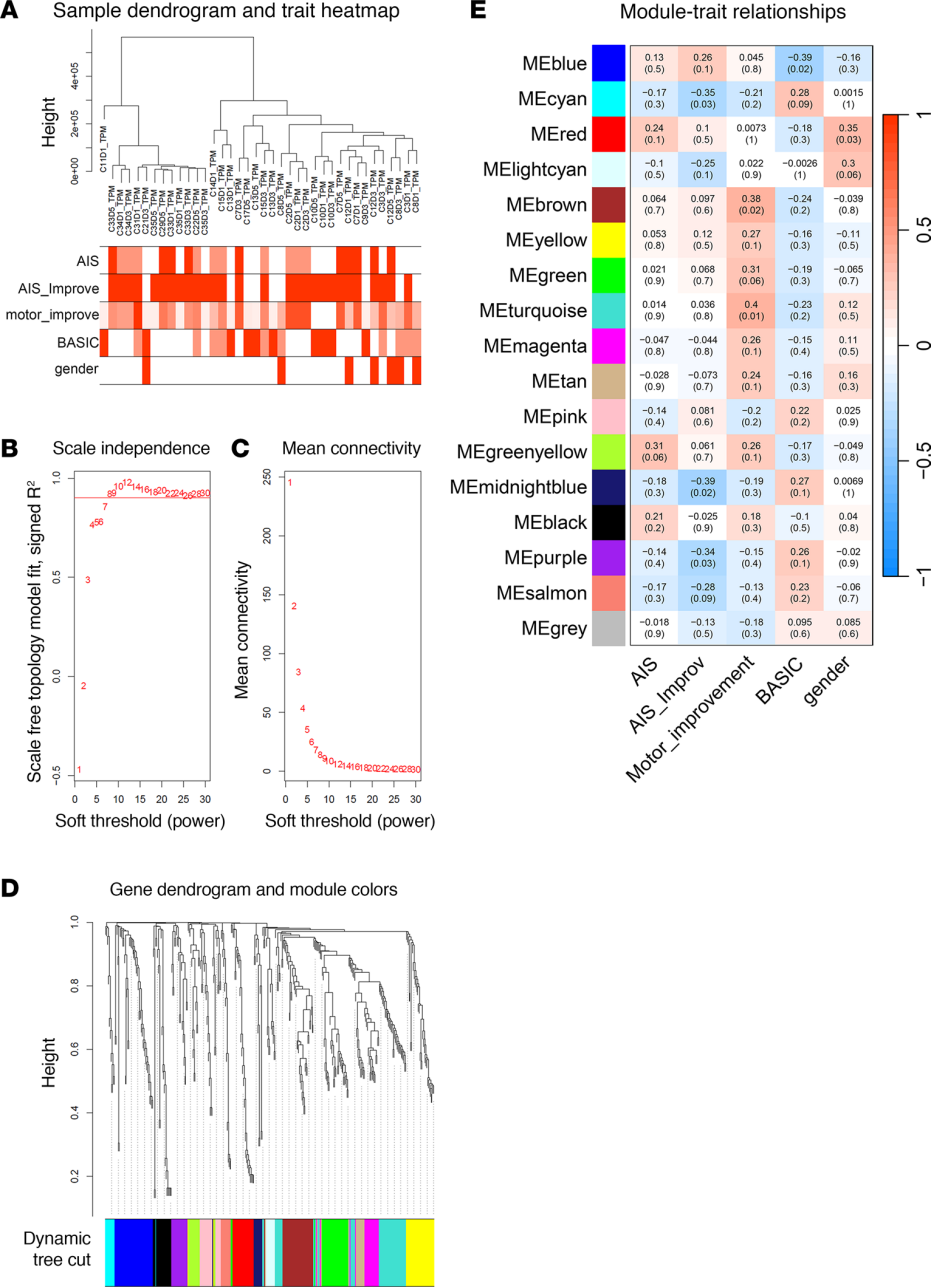
Identification and correlation of WGCNA modules across all time points. (**A**) Sample dendrogram and trait heatmap, displaying 5 traits: AIS, AIS improvement, motor improvement, BASIC, and sex. (**B**) Scale-free topology fit index (*y* axis) across different soft-thresholding powers (β) (*x* axis). (**C**) Mean connectivity (degree, *y* axis) analysis for various soft-thresholding powers (*x* axis). (**D**) Gene clustering dendrogram based on topological overlap, with assigned module colors. (**E**) Module-trait relationships, where each row represents a module eigengene (ME) and each column a trait. Correlation values (top) and *P* values (bottom) are displayed in each cell, color-coded by correlation strength.

Further, to identify coordinated gene expression networks associated with SCI severity and recovery, we applied weighted gene co-expression network analysis (WGCNA) to miRNAs with read counts ≥5 in at least 3 filtered CSF samples. This approach reduced transcriptome-wide expression variability and yielded 450 miRNAs for further analysis. Before network construction, samples were clustered and visualized via heatmap to assess the relationship between clinical traits and sample groupings (Figure 3A). For network construction, pairwise miRNA expression correlations were computed, and the resulting similarity matrix was transformed into an adjacency matrix to reflect connection strength between miRNAs. A soft-thresholding power of β = 7 was chosen to approximate scale-free network topology (scale-free *R*^2^ = 0.90; Figure 3, B and C). Hierarchical agglomerative clustering using 1-Topological Overlap Matrix as the distance metric was then performed to identify modules of co-expressed miRNAs, using the dynamic tree cut algorithm with a minimum module size of 10. This process identified 17 distinct co-expression modules (Figure 3, D and E).

We then examined correlations between module eigengenes (MEs) and clinical traits, including injury severity measures (complete vs. incomplete status, assessed by AIS grade and Brain and Spinal Injury Center [BASIC] score), recovery-related outcomes (AIS improvement and motor function improvement), and sex. At a significance threshold of *P* < 0.05, the MEblue module showed a significant negative correlation with BASIC score (*r* = –0.39; *P* = 0.02), suggesting that its constituent miRNAs are associated with more severe injury. On the other hand, the brown (*r* = 0.38; *P* = 0.02) and turquoise (*r* = 0.40; *P* = 0.01) modules were positively correlated with motor improvement. In contrast, the cyan (*r* = –0.35; *P* = 0.03), midnight blue (*r* = –0.39; *P* = 0.02), and purple (*r* = –0.34; *P* = 0.03) modules were negatively correlated with AIS improvement.

**Figure 4:**
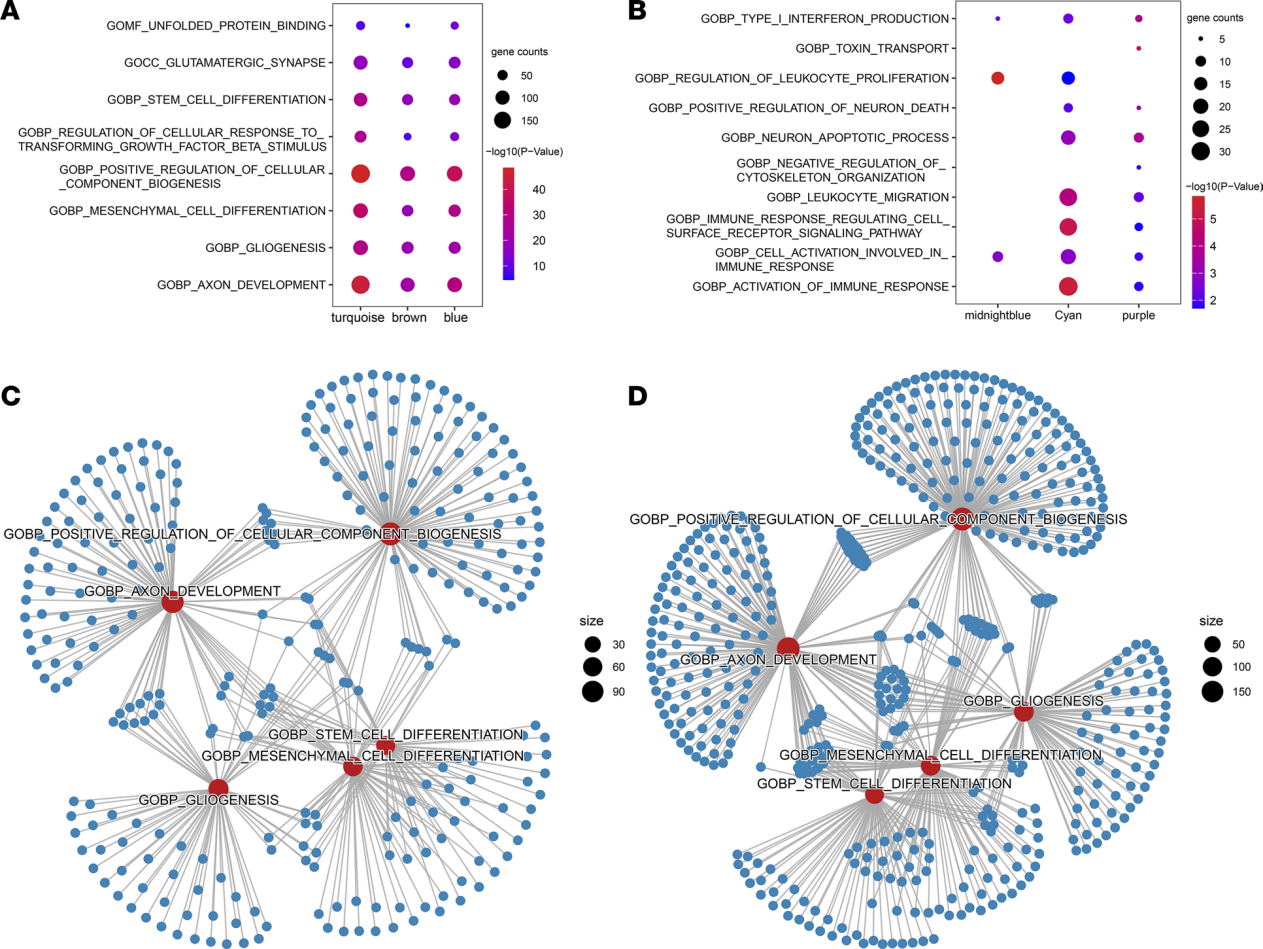
Gene set enrichment and the network of functional categories and their associated genes in WGCNA modules across all time points. (**A** and **B**) Dot plots of Gene Ontology enrichment analysis for turquoise, brown, and blue modules (**A**) and midnight blue, cyan, and purple modules (**B**). (**C** and **D**) The network in each module visualization highlights enriched functional categories and their associated genes. Functional enrichment analysis for the brown (**C**) and turquoise (**D**) modules was performed based on the target genes of hub miRNAs.

To investigate the functional roles of these modules, hub miRNAs were identified within each module by |module membership| > 0.8 and |gene significance| > 0.2 (Supplemental Table 2), and their target genes were determined by intersecting results from 2 validated miRNA-target databases: TarBase v9.0 (24) and miRTarBase v9.0 (25). Only targets present in both databases were retained for downstream analysis. Functional enrichment analysis of the miRNA target genes in each module was performed using the clusterProfiler package. As shown in Figure 4, A and B, the turquoise, brown, and blue modules were significantly enriched in pathways related to unfolded protein binding, stem cell differentiation, gliogenesis, and axon development, etc. In contrast, the midnight blue, cyan, and purple modules were enriched in immune response, neuronal apoptosis, toxin transport, and type I interferon signaling (Figure 4, C and D, and Supplemental Figure 2, A–D).

### Differential expression of exosomal miRNAs in complete versus incomplete SCI groups.

**Figure 5:**
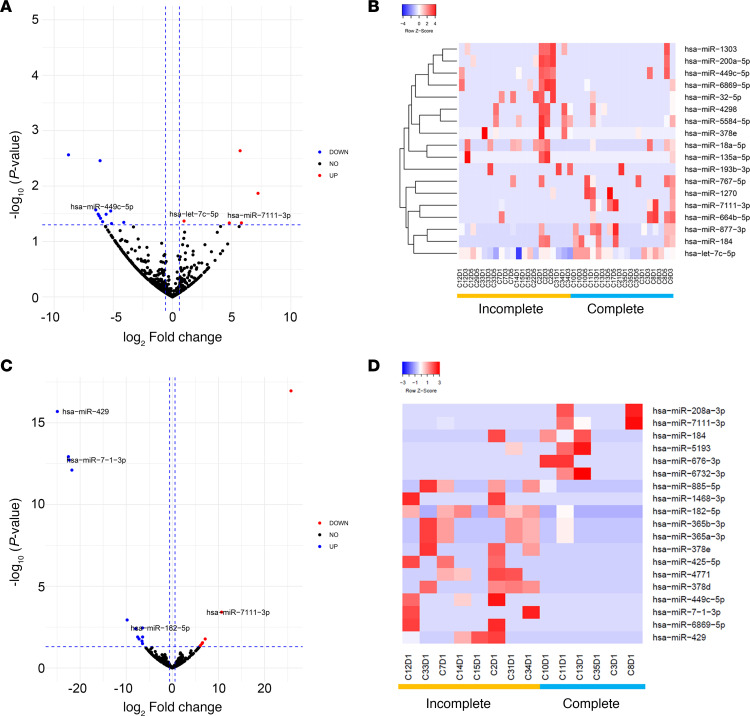
Differential gene expression analysis between complete and incomplete injuries in CSF. (**A** and **B**) Volcano plot (**A**) and heatmap (**B**) of DEGs among all patients with complete and incomplete injuries. (**C** and **D**) Volcano plot (**C**) and heatmap (**D**) of DEGs among patients (from day 1) with complete and incomplete injuries. Blue dots indicate DEGs with fold changes ≤ –1.5 and *P* value < 0.05, while red dots represent DEGs with fold changes ≥ 1.5 and *P* value < 0.05.

To identify miRNA signatures based on injury severity (complete AIS A vs. incomplete AIS B/C), we performed differential expression analysis using DESeq2 to identify significantly altered genes from days 1, 3, and 5 (Figure 5, A and B). This analysis identified 18 significantly differentially expressed genes (DEGs), including 7 miRNAs upregulated and 11 miRNAs downregulated in the complete injury group (Figure 5, A and B).

We additionally analyzed expression patterns in the acute phase of SCI at different time points both to better understand this secondary injury cascade and to potentially improve clinical decision-making. Evaluating DEGs on day 1 is clinically relevant, as SCI patients present to the hospital within 24 hours of injury for rapid evaluation and treatment. Therefore, the diagnostic utility of significant day 1 DEGs can aid in decision-making, especially if a patient has an unreliable neurological exam. Figure 5, C and D, displays 19 significant DEGs, including 6 genes upregulated in complete cases and 13 downregulated. Analysis of miRNA expression profiles revealed a severity-dependent pattern, with AIS A patients showing reduced expression of several miRNAs. On day 1 after injury, AIS A patients exhibited significantly lower levels of hsa-miR-429, hsa-miR-182-5p, and hsa-miR-7-1-3p. miR-429 has been previously shown to regulate neuronal survival. In a rat SCI model, reduced miR-429 levels were associated with increased neuronal apoptosis through activation of PTEN, which inhibits the PI3K/Akt signaling pathway — a critical axis for promoting cell survival (26). Additionally, miR-429 has been shown to suppress Notch1 signaling, which is implicated in neural injury under hypoxic conditions (27), suggesting that its downregulation may exacerbate neurodegenerative processes following SCI. Similarly, miR-182 plays a protective role in SCI by targeting the IKKβ/NF-κB pathway. Inhibition of this proinflammatory cascade by miR-182 results in reduced levels of IL-1, IL-6, and TNF, thereby suppressing inflammation and apoptosis during secondary injury (28). In our cohort, the decreased expression of miR-182 in AIS A patients is consistent with increased inflammatory damage and more severe outcomes. miR-7, particularly miR-7a, has also been reported as neuroprotective. It downregulates NF-κB signaling while upregulating *Bcl-2*, an anti-apoptotic protein, promoting cell survival after SCI. In rat models, intrathecal administration of miR-7a led to increased expression of neurotrophic factors such as *BDNF* and *NT-3*, as well as enhanced axonal regeneration and structural repair at the injury site (29). Beyond these functions, miR-7 also targets mitochondrial pathways by regulating VDAC1 and promotes antioxidative defense by inhibiting Keap1, thereby activating the Nrf2 pathway and protecting against ROS-induced toxicity (30). Together, these data highlight the dynamic and injury severity–dependent regulation of neuroprotective and inflammatory miRNAs in acute SCI, supporting their potential roles as both biomarkers and therapeutic targets.

### Differential expression of exosomal miRNAs in AIS A improvement versus non-improvement groups.

Given the variability in patient recovery outcomes, we aimed to predict recovery results based on early miRNA characteristics. Using clinical data on neurological exams recorded at 3 months after injury, we classified samples into improvement (>1 AIS grade) and non-improvement groups and identified significant DEGs between the two groups (Supplemental Figure 2, E and F). This analysis revealed only 7 significant DEGs, with 4 upregulated and 3 downregulated in the improvement group. For example, miR-449a suppresses glioma cell proliferation by downregulating PKCα expression (31) and also facilitates neurological recovery and promotes neuronal differentiation (32, 33).

Further evaluation revealed that most patients with incomplete injuries (AIS B/C) demonstrated improvement at 3 months, whereas only about half of those with complete injuries (AIS A) showed recovery. Given this strong association between initial injury severity and recovery potential, predictive modeling across the full cohort would largely reflect baseline severity differences rather than true biological signals of recovery (34). Therefore, to identify miRNAs specifically associated with neurological improvement independent of injury severity, we focused the recovery prediction analysis on patients with complete SCI, whose recovery outcomes are more variable, so our findings would be more clinically relevant. This approach allowed us to identify miRNAs potentially associated with differential recovery trajectories within the complete injury group. In day 1 samples, we identified 23 significant DEGs, with 11 upregulated in the AIS A improvement group (Supplemental Figure 3). We found an increase in certain genes associated with inflammation inhibition, including hsa-miR-375-3p (35), in the improvement groups (Supplemental Figure 3, A and B). Additionally, the AIS A improvement group exhibited enrichment in axon development, mesenchymal cell differentiation, and glutamatergic synapses (Supplemental Figure 3C), whereas the non-improvement groups were enriched in inflammatory response, autophagy, neuro-apoptotic processes, and fibroblast proliferation (Supplemental Figure 3D).

Additionally, we analyzed marker genes from days 3 and 5 and a combination of all days 1, 3, and 5 (Supplemental Figure 4). For days 1/3/5, we identified 30 significant DEGs, with 9 upregulated and 21 downregulated in the AIS A improvement group (Supplemental Figure 4, A and B). On days 3/5, we identified 42 significant DEGs, including 5 upregulated and 37 downregulated in the AIS A improvement group (Supplemental Figure 4, C and D). In AIS A patients who demonstrated neurological improvement, differential expression of specific miRNAs appeared to contribute to reduced neuroinflammation, inhibition of apoptosis, and enhanced neuroregeneration (e.g., hsa-miR-708-5p, hsa-miR-362-5p, hsa-miR-195-5p). For instance, miR-195 functions as a negative regulator of HIF-1α, a transcription factor that promotes neuroprotection under ischemic conditions by upregulating survival-related genes such as *VEGF* and *Bcl-2*. This regulation supports neuronal survival and facilitates functional recovery (36). Although miR-195 suppresses HIF-1α, its precise role may depend on timing and cellular context, potentially balancing protective and detrimental responses after injury. Another example is miR-362, which has been shown to inhibit PAX2 expression. In a rat SCI model, overexpression of miR-362 led to downregulation of the ERK/MEK and p38 MAPK signaling pathways — both of which are associated with inflammation and cell death. This suppression resulted in reduced neuroinflammation and apoptosis, ultimately leading to improved motor outcomes (37). miR-708-5p may suppress inflammation in rheumatoid arthritis by inhibiting the Wnt3a/β-catenin pathway (38). It is also significantly downregulated in glioma, and its restoration reduces glioma cell proliferation and invasion (39). These findings suggest that recovery-associated miRNAs modulate key signaling pathways involved in inflammation, apoptosis, and regeneration, highlighting their potential role in facilitating long-term recovery and serving as therapeutic targets in SCI.

### Astrocyte gap closure assay and miRNA mimic treatment to investigate the role of CSF exosome miRNAs in astrocyte activation.

**Figure 6:**
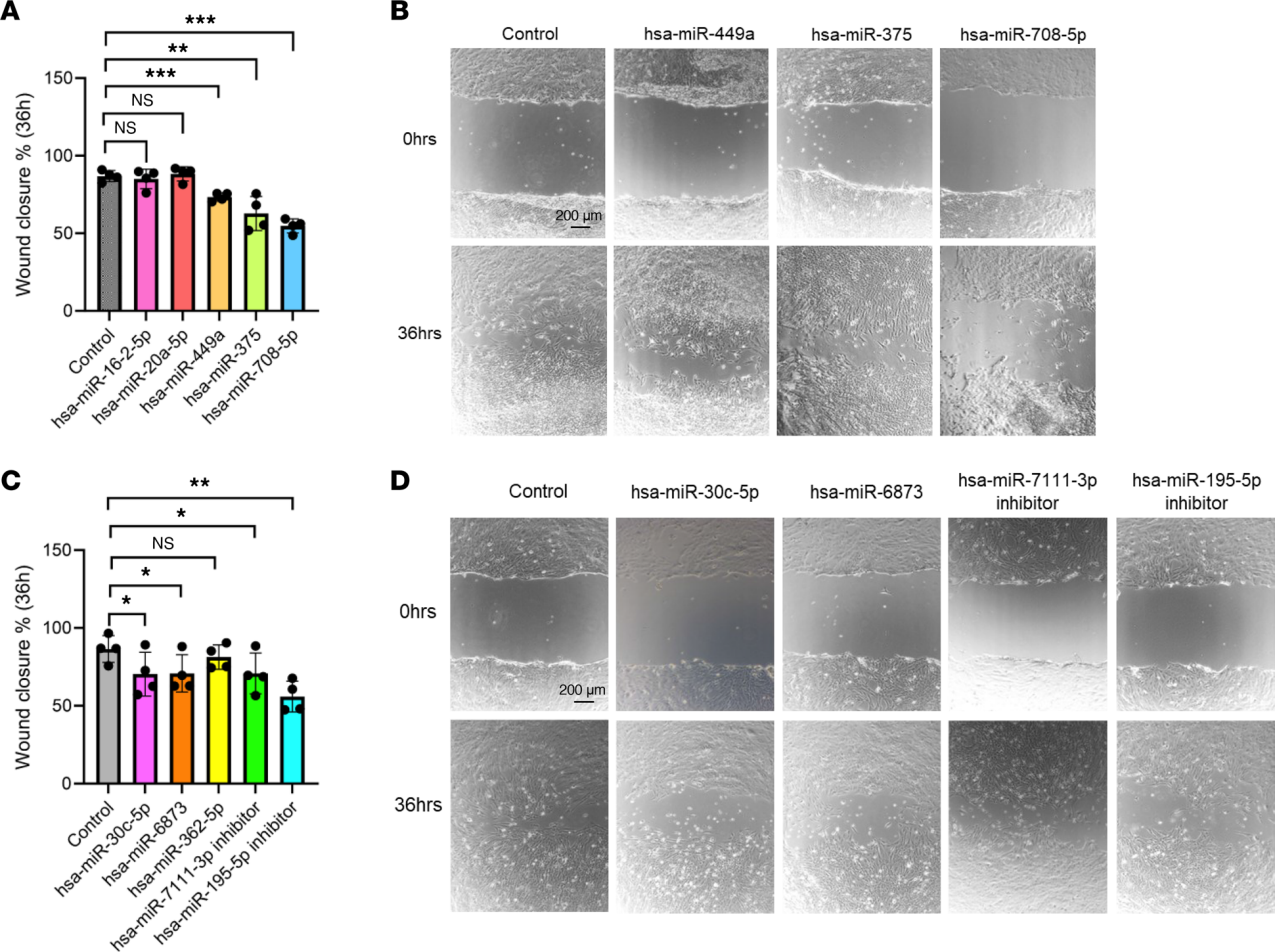
Results of astrocyte gap closure assay. Results of scratch assay at 36 hours after miRNA mimic/inhibitor treatments. (**A** and **C**) Results of gap closure measurements reveal the miRNAs that significantly inhibit astrocyte proliferation and migration in filling the gaps. Asterisks indicate significance based on unadjusted *P* values. Data are represented as mean ± SD; *n* = 4; 1-tailed independent *t* test. *P < 0.05, **P < 0.01, ***P < 0.001. Multiple-testing–adjusted *P* values (Benjamini-Hochberg, false discovery rate correction) are shown in the Supporting Data Values. In **A**, all results remained significant after correction. In **C**, miR-195-5p inhibitor retained significance after correction (adjusted *P* = 0.0085), and adjusted *P* = 0.0616 for miR-30c-5p, miR-6873, and miR-7111-3p inhibitors. (**B** and **D**) Images of scratch area from groups at 0- and 36-hour time points. Scale bars: 200 μm.

CSF exosomes originate from multiple CNS cell types, including astrocytes, microglia, and neurons. In the context of SCI, exosomes released into CSF may reflect the cellular and molecular events occurring within the CNS and facilitate long-distance intercellular communication. Emerging evidence suggests that exosomes contribute to secondary injury by delivering cell type–specific molecular cargo, such as miRNAs. Following SCI, astrocytes rapidly become reactive and undergo significant morphological and functional changes, impacting neuronal recovery (40, 41). To investigate the effects of CSF exosomal miRNAs on astrocyte behavior, we selected 10 miRNAs that were differentially expressed in the improvement group versus the non-improvement group and examined their effects using a gap closure assay after scratch to assess astrocyte proliferation and migration. Treatment of human astrocytes with mimics of hsa-miR-449a, hsa-miR-375-3p, hsa-miR-30c-5p, hsa-miR-6873, and hsa-miR-708-5p, as well as inhibitors of hsa-miR-7111-3p and hsa-miR-195-5p, resulted in a significant reduction in the rate of gap closure in comparison with the control group (Figure 6 and Supplemental Figure 5). These findings indicate that modulation of these miRNAs — either by mimic or inhibitor — suppresses astrocyte proliferation and migration.

### miRNA-mediated regulation of astrocyte secretory factors shapes neurite growth and synaptogenesis.

**Figure 7:**
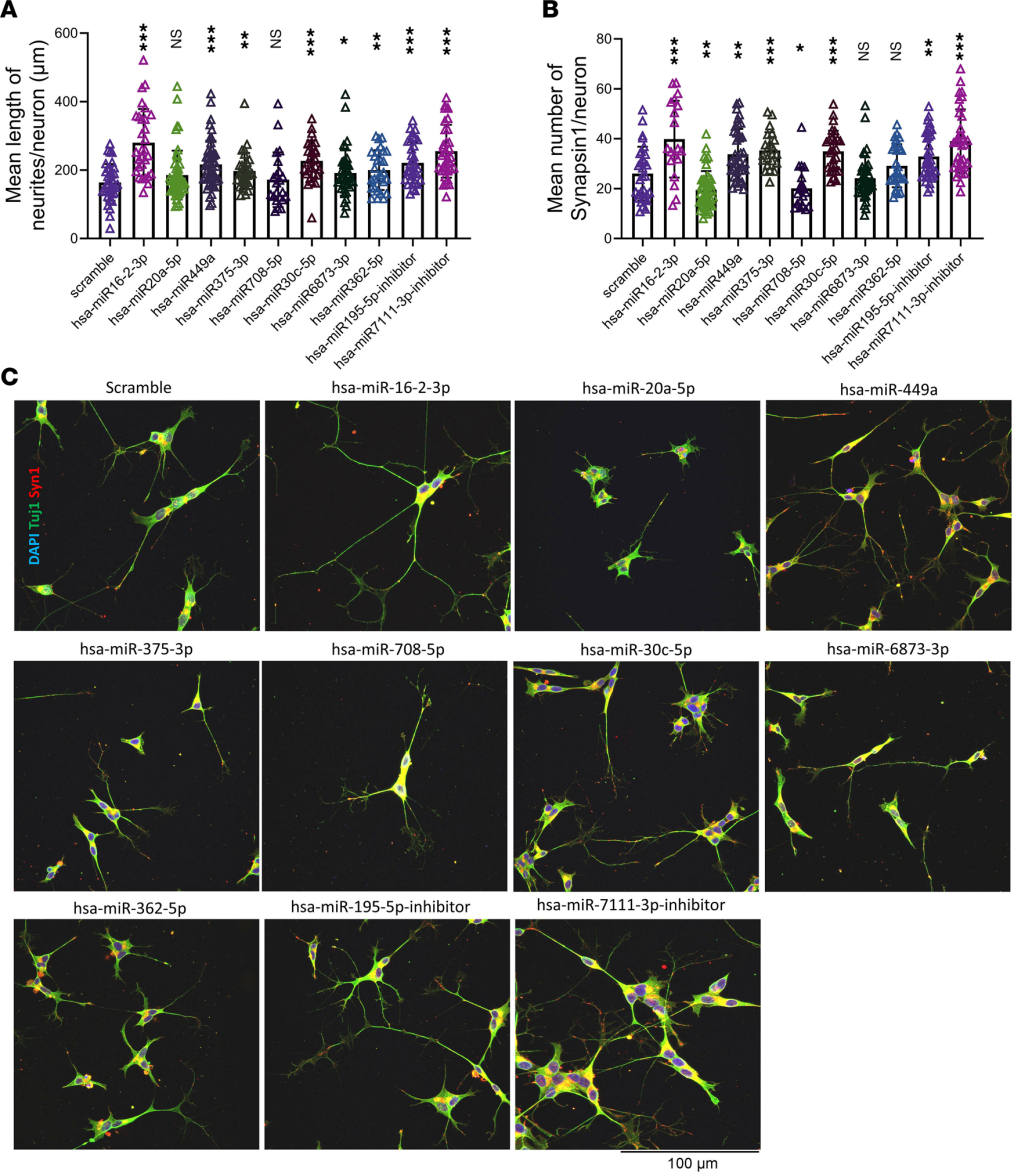
Effects of miRNA-mediated regulation on human neurite growth and synaptogenesis in a compartmentalized astrocyte-neuron coculture model. (**A** and **B**) Quantitative analysis of neurite length and synapsin-1 (Syn1) puncta per neuron in compartmentalized cocultures of injured astrocytes transfected with selected hsa-miRNA mimics or inhibitors. For neurite length and Syn1 puncta analysis, at least 20 randomly selected images per treatment group were acquired at ×40 magnification to measure the mean neurite length per neuron and the mean number of synaptic puncta per neuron. Asterisks indicate significance based on unadjusted *P* values (Benjamini-Hochberg). Multiple-testing–adjusted *P* values are shown in the Supporting Data Values. All comparisons significant by raw *P* value remained significant after correction. Data are represented as mean ± SD. *P < 0.05, **P < 0.01, ***P < 0.001 (1-tailed independent *t* test). (**C**) Representative immunofluorescence images of SH-SY5Y neurons stained for TUJ1 (green), Syn1 (red), and DAPI (blue). Scale bar: 100 μm.

Based on the observed association between specific miRNA expression patterns and patient recovery outcomes, we further examined whether astrocyte-derived factors regulated by these miRNAs could influence neuronal growth and connectivity. To this end, astrocytes were scratched and transfected with selected miRNA mimics or inhibitors and subsequently cocultured with SH-SY5Y neurons in a compartmentalized system. Neurite extension and synaptogenesis were then assessed (Figure 7). Our results demonstrated that in astrocyte-neuron coculture transfected with hsa-miR-16-2-3p, hsa-miR-449a, hsa-miR-375-3p, hsa-miR-30c-5p, hsa-miR-6873-3p, and hsa-miR-362-5p mimics, as well as hsa-miR-195-5p and hsa-miR-7111-3p inhibitors, neurite outgrowth was significantly enhanced in comparison with scramble control. Synapse formation, as quantified by synapsin-1 puncta per neuron, was also significantly increased following astrocyte transfection with hsa-miR-16-2-3p, hsa-miR-449a, hsa-miR-375-3p, and hsa-miR-30c-5p mimics, or hsa-miR-195-5p and hsa-miR-7111-3p inhibitors. In contrast, treatment with hsa-miR-20a-5p or hsa-miR-708-5p reduced synapse density despite their modest or neutral effects on neurite elongation. Importantly, 6 miRNAs — hsa-miR-16-2-3p, hsa-miR-449a, hsa-miR-375-3p, hsa-miR-30c-5p, hsa-miR-195-5p inhibitor, and hsa-miR-7111-3p inhibitor — displayed dual functions, simultaneously enhancing neurite extension and synapse formation. These results suggest that a subset of miRNAs exerts regulatory effects on astrocyte secretory programs that directly shape neuronal connectivity.

## Discussion

Recent research underscores the importance of identifying CSF and serum biomarkers that provide insight into SCI severity and can aid in predicting neurological recovery. Such biomarkers are essential for establishing objective indicators that are not captured by standard ASIA examinations or MRI. Despite growing interest, no prior studies have characterized CSF exosomal protein or miRNA biomarkers in the acute phase of SCI that are predictive of long-term outcomes. In this study, we comprehensively characterized the exosomal miRNAs and proteins in CSF and serum from SCI patients.

CSF more accurately reflects pathological changes in CNS than serum. Exosomes offer further advantages due to their cargo stability and ability to carry cell-specific signals. They can be engineered to deliver therapeutic miRNAs (42, 43) and have shown potential in SCI treatment (44–46). For example, Li et al. demonstrated that CSF-derived exosomes promoted vascular regeneration and improved motor function after SCI (47). Thus, we performed protein analysis on CSF exosomes and identified marker panels for distinguishing complete from incomplete SCI patients and improved from non-improved patients using ROC analysis. We found 15 CSF-specific and 9 serum-specific exosomal miRNAs that were consistently expressed regardless of injury severity. Using WGCNA, we identified 3 positively and 3 negatively correlated miRNA modules associated with SCI severity and recovery. Gene set enrichment analysis revealed that these modules were involved in specific biological processes. By combining clinical improvement data collected at 3 months after injury among AIS A complete SCI patients, we identified differentially expressed miRNAs between improvement and non-improvement cases. Functional tests using an astrocyte gap closure assay and astrocyte-neuron coculture demonstrated that a number of miRNAs enriched in the improvement group decreased astrocyte proliferation and migration, and promoted neurite extension and synapse formation. Given that previous knowledge of SCI pathophysiology is mostly rooted in experimental animal models, our results will help tie together preclinical data to better describe the human pathophysiology of the injury process, and highlight promising exosomal miRNA and protein biomarker candidates for improving early diagnosis, predicting long-term outcomes, and guiding therapeutic discovery in SCI.

Our findings demonstrate that protein and cytokine levels in CSF-derived exosomes are strongly associated with SCI severity and neurological recovery. Using a panel of 5 proteins, we developed a predictive model that classified injury severity with an accuracy of 0.854. A separate model, based on 4 proteins, predicted neurological improvement — defined as a gain of at least 1 AIS grade at or beyond 3 months after injury — with an accuracy of 0.813. Similarly to our approach, previous studies have sought to correlate protein biomarkers with clinical and imaging features in SCI and other neurological conditions (48). For example, Kwon et al. constructed a 6-protein model (IL-6, IL-8, MCP-1, tau, GFAP, and S100β) that predicted 6-month recovery with 83% accuracy (6), outperforming the prognostic utility of baseline ASIA grade (Concordance Index = 0.773). In addition, Skinnider et al. reported dynamic protein changes in acute SCI using total CSF protein profiling. They identified protein biomarkers associated with injury severity and recovery (49), providing valuable information. While these studies focused on whole CSF, our work specifically examines CSF-derived exosomes. CSF is a complex fluid that bathes the CNS and contains a mixture of proteins, nucleic acids, metabolites, and cellular debris released from various cell types, while exosomes are small EVs actively secreted by cells and selectively packaged with specific proteins, lipids, and RNAs, including miRNAs, reflecting the physiological or pathological state of their cells of origin. Because of this selective packaging, the molecular profile of CSF exosomes can differ substantially from that of whole CSF. This distinction is particularly important in disease contexts such as SCI, where exosomes may carry more precise information and low-abundance biomarkers that are diluted or masked in whole CSF. Therefore, analyzing CSF-derived exosomes can provide unique insights into cellular responses and disease mechanisms that are not always apparent from examining whole CSF alone.

Emerging evidence indicates that individuals with SCI are at elevated risk for developing Alzheimer’s disease (AD) compared with non-SCI populations (50, 51). Aβ is a well-established biomarker in AD, and our data suggest a link between Aβ_42_ levels and SCI severity. In particular, we found that Aβ_42_ levels in CSF-derived exosomes were lower in patients with severe injuries. Given that CSF plays a critical role in clearing waste from the CNS, including Aβ, this observation aligns with reports showing higher Aβ levels in healthy individuals relative to those with AD. Consistently, in our cohort, Aβ_42_ levels were higher in patients with incomplete injuries compared with those with complete injuries, suggesting that more effective Aβ clearance during the acute phase of SCI may support better long-term functional recovery. These findings underscore the importance of further investigating the role of Aβ in the transition from acute SCI to chronic neurodegeneration. A deeper understanding of the mechanisms could inform the development of exosome-based therapeutics targeting both SCI-related pathology and dementia risk.

miRNAs contained within CSF-derived exosomes reflect dynamic molecular changes within the CNS, making them promising biomarkers for distinguishing between complete and incomplete SCI. To identify miRNAs associated with injury severity and neurological recovery, we performed WGCNA, which revealed several modules correlated with key clinical outcomes. Functional inference was conducted by analysis of the target protein-coding genes of hub miRNAs within each module. Notably, the turquoise and brown modules were significantly associated with motor recovery. Target gene analysis of miRNAs in these modules revealed strong enrichment for pathways implicated in SCI. For example, prior studies have demonstrated that activation of the unfolded protein response can mitigate tissue damage and enhance locomotor recovery following SCI (52). Several hub miRNAs within these recovery-associated modules also appear to play roles in neuronal function. Among them, miR-449a, identified as a hub gene in the turquoise module, was enriched in patients who exhibited neurological improvement (Supplemental Figure 2F). Consistent with our findings, previous research using cerebral ischemia models showed that miR-449a upregulation promoted neurological recovery via activation of the PI3K/Akt signaling pathway (33). In contrast, the midnight blue, cyan, and purple modules were primarily associated with immune response pathways, which are generally considered detrimental to SCI recovery. These findings highlight the functional heterogeneity of miRNA modules and suggest that distinct regulatory networks contribute to either recovery or persistent dysfunction following SCI.

**Figure 8:**
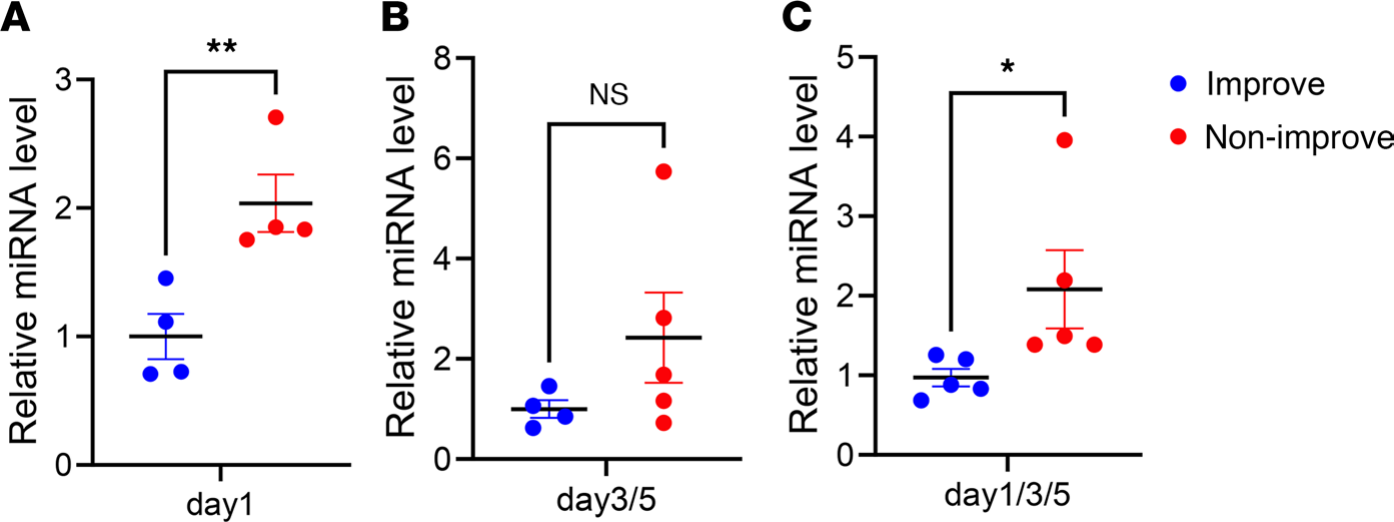
qPCR analysis of hsa-miR-7111-3p expression in patient CSF exosomes. hsa-miR-7111-3p expression level was higher in the non-improvement patient group compared with the improvement patient group. (**A**) Day 1 after injury (improvement patients, *n* = 4; non-improvement patients, *n* = 4). (**B**) Day 3/5 after injury represents pooled samples from days 3 and 5 (improvement patients, *n* = 4; non-improvement patients, *n* = 5). (**C**) Day 1/3/5 after injury represents pooled samples from days 1, 3, and 5 (improvement patients, *n* = 5; non-improvement patients, *n* = 5). Results are expressed as mean ± SEM for each group. *P < 0.05, **P < 0.01 (1-tailed independent *t* test).

We observed that most patients with incomplete SCI showed neurological improvement within 3 months, whereas approximately half of the patients with complete injuries also demonstrated measurable recovery. To further investigate the molecular basis of these outcomes, we analyzed CSF exosomes collected from complete SCI cases at days 1, 3, and 5 after injury. This analysis identified miRNAs with significantly altered expression in patients who showed neurological improvement compared with those who did not. Functional studies indicated that these improvement-associated miRNAs exert important effects on astrocyte biology. Specifically, miRNAs can influence astrocyte proliferation and migration and, through astrocyte-derived secretory factors, modulate neurite outgrowth and synaptogenesis. We separately tested mimics for 8 miRNAs positively correlated with patient improvement. When astrocytes were treated with mimics of 5 of these miRNAs, cell proliferation and migration were inhibited, suggesting a regulatory role in astrocyte activation. Conversely, we inhibited 2 miRNAs that were negatively associated with patient improvement and also observed decreased astrocyte proliferation and migration. Of the 10 tested, 8 miRNAs demonstrated functional roles in promoting neurite extension, and 8 miRNAs in synapse formation in astrocyte-neuron coculture. Combining the results from both astrocyte gap closure assays and astrocyte-neuron coculture, we found that mimics of hsa-miR-375 and hsa-miR-30c-5p suppressed astrocyte proliferation and migration, and enhanced neurite growth and synaptogenesis. Also, inhibitors of hsa-miR-7111-3p and hsa-miR-195-5p produced similar effects, suggesting that these miRNAs may contribute to balancing glial activity and promote neuronal regeneration. Mechanistically, miR-375-3p has been reported to regulate neurogenesis and motor neuron development through modulation of PAX6 and CCND2, and to suppress p53-mediated apoptosis (53). However, the roles of many of the other miRNAs identified in our study remain poorly characterized, with limited prior research available regarding their impact on neuronal development or recovery. For example, hsa-miR-7111-3p was specifically enriched in non-improvement cases and, to our knowledge, has not been previously reported in the SCI literature. Consistent with these findings, qPCR validation demonstrated that hsa-miR-7111-3p expression was significantly elevated in non-improvement cases, at day 1 and days 1/3/5 after injury (Figure 8, A and C), supporting its potential as a robust biomarker predictive of SCI recovery outcomes. Although the comparison at days 3/5 alone did not reach statistical significance (Figure 8B), the expression trend remained consistent with the sequencing results. Together, these findings suggest that improvement-associated miRNAs may contribute to neurological recovery after SCI by regulating astrocyte activation and the release of astrocyte-derived factors that influence neuronal regeneration. By fine-tuning astrocyte secretory activity, these miRNAs may facilitate neuronal repair and functional recovery after SCI, highlighting their potential as therapeutic targets for enhancing repair mechanisms following SCI.

Despite its strengths, our study has limitations. Attrition is a common challenge in SCI research owing to patient mobility limitations and follow-up barriers. However, our cohort had the benefit of clinical follow-up through 3 months, which is the time with the most rapid rate of motor recovery (54). Although CSF was collected on days 1, 3, and 5, some samples were excluded because of insufficient volume or contamination with blood. This is due to the inherent nature of SCI, with traumatic bleeding into the CSF occasionally causing issues with drainage through the lumbar catheter. CSF was obtained only during the initial hospitalization and not in the clinic at follow-up, as obtaining CSF at follow-up is not the standard of care. Lumbar puncture has risks such as bleeding, infection, CSF leak, and nerve injury; therefore, it is not medically/ethically appropriate to obtain in these patients without any diagnostic or therapeutic benefit. For statistical analysis, to control type I error, multiple-testing correction was applied using the Benjamini-Hochberg false discovery rate method. The adjusted *P* values demonstrated that the majority of findings remained significant after correction; for example, 27 of 30 comparisons in the Supporting Data Values file (corresponding to Figure 6, A and C, and Figure 7, A and B) were consistent between raw and adjusted analyses. Therefore, adjusted *P* value analyses support our overall conclusions. Future studies with larger sample sizes will further enhance the rigor of the study. Finally, the experiments were primarily designed to investigate the effects of miRNAs on astrocytes, and astrocyte-neuron interactions. Although the effects on microglia are outside the scope of the current study, this could be an interesting area for future study.

In conclusion, our study presents an extensive investigation of the human exosome in the CSF and serum of patients with acute SCI. We identified miRNA and protein biomarkers in CSF-derived exosomes that could potentially improve early diagnosis and predict outcome and treatment of SCI. These findings provide a foundation for future mechanistic investigations into targeted therapies and personalized medicine approaches for SCI recovery.

## Methods

### Sex as a biological variable.

Sex was not evaluated as a key biological variable. Both male and female human participants were used in this study.

### Study design and patient enrollment.

This is a single-center prospective study of acute SCI patients presenting to our level I trauma center (Memorial Hermann Hospital–Texas Medical Center, Houston, Texas, USA). Patients presenting to the Memorial Hermann Emergency Department who suffered an acute traumatic SCI were screened for the study. Patients were included if they met the following criteria: (a) aged 18–80 years; (b) AIS grade A, B, or C SCI; (c) injury within 24 hours of presentation. Patients were excluded if they had SCI from penetrating mechanism, injury below L1, concomitant head injury with a clinically significant abnormality on brain CT, traumatic injuries that preclude spine surgery within 24 hours of presentation, preexisting neurological or mental disorder that would preclude accurate evaluation and follow-up (i.e., Alzheimer’s disease, Parkinson’s disease, unstable psychiatric disorder with hallucinations and/or delusions, or schizophrenia), pregnancy, or prior history of SCI, or were prisoners. Informed consent was obtained for all patients. For patients who were unable to sign for themselves because of injury, a witness was present throughout the consenting process, and the consent was then signed on the patient’s behalf. Patients incapable of providing consent were enrolled via a legally authorized representative in accordance with Texas law. Once the patient improved and could reliably consent, formal consent was obtained.

### Neurological classification of SCI.

To determine the severity of neurological impairment after SCI, AIS grade was obtained from ASIA examination in accordance with International Standards for Neurological Classification of Spinal Cord Injury guidelines (55). AIS A was defined as complete SCI, with no preservation of motor or sensory function below the neurological level of injury. Incomplete SCI included patients with AIS B, C, and D spinal cord injury. AIS B was defined as preservation of sensory but not motor function below the injury level. AIS C was defined as motor incomplete, with more than half of key muscle groups below the level of injury having a muscle grade of less than 3 (i.e., unable to move against gravity). AIS D was defined as motor incomplete, with more than half of key muscle groups having a muscle grade of 3 or more (i.e., able to move against gravity). Motor scores were determined during each ASIA exam by evaluation of 5 key muscle groups in each extremity and scored on a standard strength scale ranging from 0 to 5, with a maximum score of 25 for each extremity and 100 for the total motor score.

ASIA examinations were conducted by neurosurgical house staff. AIS score was obtained immediately upon initial examination in the Emergency Department. Subsequent examinations were completed daily during hospitalization, prior to discharge, and at follow-up in clinic by the neurosurgical resident and attending physician. Our cohort had clinical follow-up through 3 months (54). Based on clinical follow-up data, patients were classified as improved if they had improvement in neurological function by ≥1 AIS grade (e.g., AIS A initially, conversion to AIS B at follow-up). Patients who did not have AIS grade conversion at follow-up were classified as the non-improved subgroup.

In addition to clinical examination, known radiographic biomarkers for acute SCI were evaluated on T2-weighted MRI. The Brain and Spinal Injury Center (BASIC) score describes 5 distinct patterns of intramedullary signal abnormality on T2 MRI, which grade severity of acute SCI using a 0–4 point ordinal scale (56). Studies have shown correlation between the BASIC score and AIS grades on initial evaluation, as well as long-term improvement in AIS grade at follow-up (56, 57).

### Intervention.

All patients underwent surgical decompression and stabilization within 24 hours of SCI. At the time of surgical intervention, an intrathecal catheter was placed using standard antiseptic technique. The catheter was inserted at L3/4 or L4/5 and advanced 15–20 cm from the entry point. Postoperative care for all patients included monitoring of mean arterial pressure (MAP) and spinal cord perfusion pressure (SCPP) with targeted goals of MAP ≥ 85 mmHg and SCPP ≥ 65 mmHg for 120 hours. Blood pressure was augmented as needed to meet the MAP goal, and 10 cc of CSF was drained hourly to reduce the intrathecal pressure and help maintain the SCPP goal. Catheters were kept in place for 120 hours unless they were malfunctioning and unable to be troubleshot.

### Biospecimen collection and processing.

Approximately 10 mL CSF was collected at the time of lumbar drain placement, on days 3 and 5. A 10 mL blood sample was obtained at the time of each CSF collection. CSF samples were then collected according to our institution’s intensive care unit protocol. Up to 10 mL fresh CSF sample was drawn via the Buretrol port (Integra LifeSciences Corporation) into a collection bag using a sterile syringe. CSF samples were processed within 1 hour of collection. Samples were centrifuged at 2,000*g* for 10 minutes, and the supernatant was aliquoted in 2.0 mL tubes and stored in a –80°C freezer. At the time of CSF collection, peripheral blood was obtained from the arterial line or via standard sterile venipuncture and collected into a 10 mL sterile tube. Blood was allowed to clot for 30–90 minutes at room temperature and then centrifuged at 2,000*g* for 15 minutes. The serum supernatant was then divided into multiple aliquots in new 2.0 mL tubes and immediately stored in a –80°C freezer.

### Patient data collection.

Collected data were housed in a Research Electronic Data Capture (REDCap) database. REDCap is fully compliant with Health Insurance Portability and Accountability Act security standards for protection of personal health information and data. The methods and protocols used in this study were approved by the Institutional Review Board at Memorial Hermann Hospital.

### Exosome isolation and processing.

Exosomes were isolated from patient CSF samples. CSF exosome isolation was performed according to the manufacturer’s instructions, with minor modifications, using the miRCURY Exosome Cell/Urine/CSF Kit (QIAGEN, catalog 76743). Briefly, CSF samples were completely thawed on ice, and 1 mL aliquots of CSF (total volume 2 mL) were centrifuged at 3,000*g* for debris removal at 4°C. The cleared CSF was then thoroughly mixed with 500 μL of Precipitation Buffer B included in the miRCURY Exosome Cell/Urine/CSF Kit and vortexed briefly for 30 seconds to 1 minute, followed by incubation at 4°C overnight. After incubation, samples were centrifuged at 10,000*g* for 30 minutes at 20°C. Thereafter, supernatant was discarded gently without disturbing the exosome pellet.

### NanoSight particle tracking analysis.

CSF exosomes were analyzed using a NanoSight NS300 Instrument (Malvern). Samples were diluted 1:100 with sterile water to gain an optimal reading range from 20 to 150 particles per frame. Three videos of 60 seconds were recorded using the green laser module, 532 nm, and the relative data were analyzed using NanoSight NTA Software 3.4 (Malvern). The instrument settings were optimized and remained constant between samples. Data collected on the particles consisted of average size (nm), mode, size distribution (D values: D10/D50/D90), and particle concentration (particles/mL).

### Characterization of exosomes by cryo-EM.

The morphology of exosomes was examined using cryogenic electron microscopy (cryo-EM) at the Cryo-EM Core Facility at UTHealth. For sample preparation, Quantifoil EM grids were glow-discharged (15 seconds, 30 mA) to enhance sample adhesion. A 3 μL aliquot of the aqueous sample solution was applied to a Quantifoil grid (200 mesh, R2/1), blotted for 3–4 seconds, and plunge-frozen into precooled liquid ethane using a Vitrobot Mark IV (Thermo Fisher Scientific). The samples were analyzed using a 200 kV Cryo-TEM Glacios microscope (Thermo Fisher Scientific) equipped with a Falcon 4i camera and a Selectris X energy filter. Images were captured at a magnification of ×79,000 with a pixel size of 1.58 nm and a defocus of –3 μm. The total accumulated electron dose per image was approximately 40 e^–^/Å^2^.

### Total exosome RNA and protein isolation.

The Invitrogen Total Exosome RNA & Protein Isolation Kit (catalog 4478545) was used for both CSF RNA and protein isolation in accordance with manufacturer protocols. Exosome pellets were resuspended in 100 μL of exosome resuspension buffer per 1 mL of CSF sample and incubated at room temperature for 10 minutes. For each patient, CSF samples yielded a total of 200 μL of exosome suspension in resuspension buffer. The eluted RNA was quantified by a Thermo Fisher Scientific NanoDrop Spectrophotometer, and RNA integrity number (RIN) was determined by RIN assay in the Cancer Genomics Center core facility, UTHealth. The isolated protein was resuspended in 200 μL exosome resuspension buffer.

### cDNA synthesis and miRNA RT-qPCR.

Candidate miRNAs were subsequently validated by qPCR in an independent cohort of CSF exosome samples, comprising 8 samples from 5 SCI-improved patients and 9 samples from 5 SCI-non-improved patients. cDNA synthesis and miRNA RT-qPCR were performed using the miRCURY LNA miRNA PCR Starter Kit (QIAGEN, catalog 339320). Approximately 100 pg of total RNA isolated from CSF-derived exosomes was reverse-transcribed in 10 μL reactions using a thermal cycler. An RNA spike-in control from the miRCURY LNA kit was added before cDNA synthesis for quality control and normalization. The resulting cDNA was diluted 1:30 and used as input for 10 μL qPCRs. Amplification was carried out on a real-time PCR system (C1000 Thermal Cycler, Bio-Rad). miRNA from each patient sample was analyzed in 3 technical replicates. The primer sequences specific for hsa-miR-7111-3p were obtained from catalog YP02110682.

### miRNA library preparation and sequencing.

miRNA libraries were prepared and sequenced at the University of Houston Seq-N-Edit Core per standard protocols. miRNA libraries were prepared with the QIAseq miRNA library kit (QIAGEN) using 5 μL of the extracted miRNA sample. Libraries were produced by sequential ligation of adapters to the 3′ and 5′ ends of miRNAs. This was followed by reverse transcription into cDNA and subsequent ligation of sample indexes and sequencing adapters. The size selection for libraries was performed using SPRIselect beads (Beckman Coulter). Library purity was analyzed using DNA HS1000 tape on a Tapestation 4200 (Agilent) and quantified with a Qubit Fluorometer (Thermo Fisher Scientific). The prepared libraries were pooled and sequenced using a NextSeq 2000 (Illumina), generating 76 bp single-end reads.

### Sequencing data analysis.

The sequencing data preprocessing involved removing adapters, indexes, and low-quality reads using Cutadapt (58). Small RNAs ranging from 17 to 40 nucleotides were then selected for further analysis. These small RNAs were aligned to mature human miRNA sequences from miRBase (59) using Bowtie (60). The aligned reads were sorted and counted for each miRNA using SAMtools (61). miRNAs with read counts less than 5 in more than 10 samples were discarded, as these were unlikely to yield stable and robust results. Raw counts were normalized for sequencing depth using the transcripts per million method (transcripts per million = mapped read count/total reads × 10^6^). Samples failing to cluster with others from the same organ type in hierarchical clustering analysis were removed.

Filtered CSF samples underwent weighted gene co-expression network analysis (62). Briefly, a co-expression similarity measure was calculated by raising of the absolute correlation coefficient between genes to a selected power. An adjacency matrix was constructed using a soft-thresholding procedure and served as the basis for generating a hierarchical clustering of genes. Module-trait relationships were computed using Pearson’s correlation between module eigengenes and SCI traits. An eigengene, defined as the first principal component of a given module, represents the miRNA expression profiles within that module. Modules with a *P* value less than 0.05 were selected for further analysis. Potentially biologically interesting modules underwent downstream analysis, and networks for miRNAs in these modules were visualized using Cytoscape 3.10 (63). Hub genes were identified in the module filtered by |module membership| > 0.8 and |gene significance| > 0.2.

DEG analysis was performed using DESeq2 (64) with *P* value < 0.05 and |fold change| > 1.5. Volcano plots were generated using ggplot2 (https://ggplot2.tidyverse.org/index.html), and heatmaps were created using heatmap.2 (https://talgalili.github.io/gplots/). miRNA target genes were identified using multiMiR (65) packages, referencing the TarBase (v9.0) (24) and miRTarBase (v9.0) (25) databases. Genes present in both databases were selected as miRNA target genes. Functional enrichment analyses of these target genes were conducted using the clusterProfiler package (version 4.8.3) (66) and the MSigDB R database (67). A *P* value threshold of less than 0.05 was considered statistically significant.

### Multiplex immunoassay.

Concentrations of cytokines/chemokines (CCs), neuronal proteins, and exosome protein markers were measured using premixed multiplex assays in CSF exosome protein isolate (Thermo Fisher Scientific, catalog LHC6003M, EPX180-15837-901, and EPX060-15845-901). The CCs measured in the assay were EGF, eotaxin (CCL11), FGF-2, G-CSF (CSF-3), GM-CSF, HGF, IFN-α, IFN-γ, IL-1β, IL-1RA, IL-2, IL-2R, IL-4, IL-5, IL-6, IL-7, IL-8 (CXCL8), IL-10, IL-12/IL-23p40, IL-13, IL-15, IL-17A (CTLA-8), IP-10 (CXCL10), MCP-1 (CCL2), MIG (CXCL9), MIP-1α (CCL3), MIP-1β (CCL4), RANTES (CCL5), TNF-α, and VEGF-A. The neuronal proteins measured in the assay were amyloid-β_1–42_, BDNF, CNTF, FGF-21, GDNF, GFAP, kallikrein-6 (KLK6), MIF, NCAM-1, NF-H, NGF-β, NRGN, S100B, tau (total), tau (p-T181), TDP-43, UCHL1, and YKL-40 (CHI3L1). The exosome proteins measured in the assay were CD9, CD63, CD81, CYT-C, SYN-1, and VLA-4. Briefly, for the CC assay, 50 μL CSF exosome isolate was thawed completely and diluted with the same volume of provided assay buffer. For the neuronal protein assay, 25 μL CSF exosome isolate was thawed completely and diluted with the same volume of provided assay buffer. For the exosome marker assay, 25 μL CSF exosome isolate was thawed completely and diluted with the same volume of provided assay buffer. The assays were performed blindly and in duplicates on the Bio-Plex 200 suspension array system (Bio-Rad Laboratories). All assays were performed according to the manufacturer’s protocol and are reported as picograms per milliliter.

### Astrocyte gap closure assays and miRNA mimic treatment.

Human astrocytes (ATCC SVG p12, catalog CRL-8621) were cultured in 24-well plates at a concentration of 5 × 10^4^ cells per well and incubated until they reached approximately 80%–90% confluence. A scratched gap wound was created in the cell monolayer using a 200 μL sterile pipette tip, guided by a sterile ruler. Detached cells were removed by changing of the medium to EMEM (Corning, 10-009-CV) supplemented with 10% fetal bovine serum (FBS; Gibco, A5670201) without antibiotics, followed by treatment with miRNA mimics/inhibitor (Supplemental Table 3). Transfection complexes of miRNAs were prepared individually for each miRNA. Specifically, 2.5 μL of 5 μM miRNA mimic (12.5 pmol) was mixed with 25 μL of Opti-MEM (Gibco, 31985-062). In a separate tube, 1.5 μL of Lipofectamine (RNAiMAX, Thermo Fisher Scientific, 13778075) was combined with 25 μL of Opti-MEM. The miRNA mimic solution and the transfection reagent solution were then mixed at a 1:1 ratio and incubated for 15 minutes at room temperature to form transfection complexes. Scramble group served as a control group.

The transfection complexes were added directly to the culture medium containing the cells, ensuring even distribution. The cells were then incubated at 37°C in a CO_2_ incubator for 36 hours. Images were captured from selected ROIs using a Zeiss inverted microscope with a ×4 objective every 12 hours up to 36 hours. The same imaging zone was used for each well throughout the experiment. Scratch areas were determined using the ImageJ (NIH) macro. The percentage of gap closure was calculated using the formula: gap closure % = ((A_t0_ – A_t1_)/A_t0_) × 100, where A_t0_ is the scratch area at time 0 after scratch and A_t1_ is the scratch area at time point 36 hours after the scratch.

### Neuronal differentiation of SH-SY5Y cells.

Human SH-SY5Y cells (ATCC CRL-2266) were maintained in DMEM/F12 medium (Gibco, 11320033) supplemented with 10% heat-inactivated FBS (hiFBS; Gibco BRL, A5670201), 1% GlutaMAX (Gibco, 35050061), 1% non-essential amino acids (MEM, Gibco, 11140050), and 1% penicillin/streptomycin (Pen/Strep) (Gibco, 10378016). Cells were cultured at 37°C in a humidified incubator with 5% CO_2_.

For neuronal induction, SH-SY5Y cells were trypsinized and seeded at a density of 1,500 cells per chamber onto the center chambers of 1:30 diluted Matrigel-coated (Corning, CLS356231) 2-well coculture μ-slides (μ-Slide 9 × 2 well, ibidi GmbH). Differentiation was initiated by culturing of the cells for 4 days in induction medium A, consisting of DMEM (Gibco, 11965092) supplemented with 2.5% hiFBS, 1% GlutaMAX, 1% B27 supplement (Gibco, 17504044), 1% Pen/Strep, and 10 μM retinoic acid (Sigma-Aldrich, R2625). The cultures were then maintained for an additional 3 days in induction medium B, which contained Neurobasal medium (Gibco, 21103049) plus DMEM (1:1) supplemented with 1% hiFBS, 1% GlutaMAX, 1% Pen/Strep, 20 mM KCl, 1% B27 supplement, and 10 μM retinoic acid (Sigma-Aldrich, R2625). Media were refreshed daily. On day 7, the cells were switched to neurite growth medium composed of Neurobasal medium supplemented with 1% hiFBS, 1% L-GlutaMAX, 1% Pen/Strep, and 1% N2 supplement (Gibco, 17502048).

### Coculture of neurons and astrocytes.

On day 5 of neuronal differentiation, human astrocytes (SVG p12, ATCC; 1,000 cells per well) were seeded into the peripheral chambers of coculture μ-slides using Eagle Minimum Essential Medium (MEM; Sigma-Aldrich, M4655) supplemented with 10% FBS. After 2 days of culture, astrocytes were mechanically injured by scratching with a 10 μL pipette tip. On day 8 of neuronal culture, astrocytes in the peripheral chambers were treated with miRNA mimics at a final concentration of 25 nM. Transfection complexes were prepared individually for each miRNA. Specifically, 0.35 μL of 5 μM miRNA mimic (1.75 pmol) was mixed with 3.5 μL of Opti-MEM. Separately, 0.21 μL of Lipofectamine RNAiMAX was combined with 3.5 μL of Opti-MEM. The 2 solutions were then mixed at a 1:1 ratio and incubated for 15 minutes at room temperature to form transfection complexes. The scramble group served as a control. Complexes were added directly to the culture medium in each well (70 μL total), ensuring even distribution. On day 9 of neuronal culture, 300 μL of neurite growth medium was gently added to each well, exceeding the standard 70 μL capacity. This increase in volume allowed the medium to overflow the inter-chamber barriers, facilitating the diffusion of soluble factors between compartments while maintaining physical separation of cell populations and preventing direct cell-cell contact. After the addition of excess neurite growth medium, the cocultures were maintained for an additional 2 days without medium exchange, such that neurons were differentiated in neurite growth medium for a total of 4 days (days 7–11, no medium change). All hsa-miRNAs used in this study were synthesized by GenScript (Piscataway, New Jersey, USA). Details of each miRNA are presented in Supplemental Table 3. A precise flowchart of cell culture is shown in Supplemental Figure 6.

### Fixation, immunocytochemistry, image analysis, and statistical evaluation.

On day 11 of neuronal culture, cells were fixed with cold 4% paraformaldehyde for 10 minutes at room temperature. After fixation, immunocytochemistry was performed to assess neuronal maturation. Cells were permeabilized using 0.3% Triton X-100 in 1% bovine serum albumin, then incubated overnight at 4°C with primary antibodies targeting neuronal markers: chicken anti–Tuj-1 (Sigma-Aldrich, AB9354) and rabbit anti–synapsin-1 (Abcam, ab254349).

After primary antibody incubation, cells were washed 5 times for 5 minutes each with phosphate-buffered saline containing 0.25% Tween 20. Secondary antibody incubation was carried out for 2 hours at room temperature using goat anti-chicken IgY Alexa Fluor 488 (Invitrogen, A11039) and goat anti-rabbit Alexa Fluor 594 (Invitrogen, A11037). Nuclei were counterstained with DAPI (5 μM) for 5 minutes. Finally, slides were mounted and sealed for imaging.

Neuronal maturation was evaluated by quantification of neurite outgrowth and the number of synapsin-1 puncta per cell using ImageJ. For neurite analysis, images were processed using the Skeletonize function to measure total neurite length. Synapsin-1 puncta were quantified by first generating regions of interest (ROIs) based on Tuj-1–positive neurites (green channel), followed by particle analysis to count red fluorescent puncta (synapsin-1) within these ROIs. To normalize the data, the total number of puncta was divided by the number of nuclei per image, which were counted using ImageJ’s particle counting function after thresholding of the DAPI channel.

### Statistics.

Data were presented as mean ± SEM or SD. Two-group comparisons were performed using 1-tailed independent *t* test. ROC curve analysis was performed to evaluate the diagnostic performance of candidate protein biomarkers using the R package pROC (68). Briefly, ROC analysis was first conducted for each gene individually, and candidates with AUC significantly greater than 0.5 were retained for further analysis. Subsequently, logistic regression models were used to construct different combinations of the candidate biomarkers. Finally, combinations with optimal diagnostic performance (i.e., high AUC) were selected and reported.

For analyses involving repeated measurements across multiple days, the average value per subject was used to avoid inflation of type I error. To account for multiple comparisons, *P* values were additionally adjusted using the Benjamini-Hochberg false discovery rate procedure. Both raw and adjusted *P* values are reported (Supporting Data Values). The adjusted *P* values were used to assess the robustness of the findings following multiple-testing correction. For clarity, the sample size for each analysis is indicated in the figure legends and relevant sections, where applicable. *P* values less than 0.05 were considered statistically significant.

### Study approval.

The study protocol was approved by the UTHealth Houston Committee for the Protection of Human Subjects in Houston, Texas (IRB number HSC-MS-22-0391). All experiments were conducted in accordance with institutional guidelines. Written informed consent was obtained for all patients in the study.

### Data availability.

Sequencing data were deposited in the NCBI’s Gene Expression Omnibus database under accession number GSE305596. Proteomics data in human samples are included in Supplemental Table 4. Values for all data points in graphs are reported in the Supporting Data Values file.

## Author contributions

JSW, HW, JJM, and JQW conceived the project. JSW and DLS collected human samples and wrote the manuscript. HW performed data analyses and wrote the manuscript. JSW, DLS, FHM, CCL, and JR performed the experiments and wrote the manuscript. JZ and JRW collected human samples. HW and WL performed statistical analyses. All authors reviewed the final manuscript.

## Conflict of interest

The authors have declared that no conflict of interest exists.

## Funding support

This work is the result of NIH funding, in whole or in part, and is subject to the NIH Public Access Policy. Through acceptance of this federal funding, the NIH has been given a right to make the work publicly available in PubMed Central.

- NIH R01 NS088353 (to JQW).
- Amy and Edward Knight Fund (to JQW).

## Supplementary Material

Supplemental data

Supplemental Table 4

Supporting data values

## Version 1 06/23/2026

In-Press Preview

## Version 2 08/10/2026

Electronic publication
