# Supplementary material for: The investigation of human cerebrospinal fluid exosome in spinal cord injury
Source: JCI Insight. 2026 Jun 23;11(15):e205134. doi: 10.1172/jci.insight.205134 (PMC13463619; doi:10.1172/jci.insight.205134)
Supplement: Supplementary file 1 [file jciinsight-11-205134-s018.pdf]

Supplemental material

Supplemental Table **1 Clinical characteristics of study participants.** NIL = neurological injury level. C = cervical; T = thoracic; L = lumbar; AIS = ASIA Impairment Score. A = motor and sensory complete SCI; B = motor complete and sensory incomplete SCI; C = Motor incomplete SCI (more than half of key muscle functions below the NIL have a muscle grade less than 3); D = Motor incomplete SCI (at least half of key muscle functions below the NIL have a muscle grade of 3 or greater); Δ = change; UEMS = upper extremity motor score; LEMS = lower extremity motor score; TMS = total motor score; LT = light touch sensation; PP = pin prick sensation. * Included patients had >3 month follow up (range 3-6 months)

Patient ID	**Sex**	**Age**	**NIL**	**Baseline AIS**	**Follow-up AIS***	**Follow-up NIL***	**ΔUEMS**	**ΔLEMS**	**ΔTMS**	**ΔLT**	**ΔPP**
1	M	20	L1	C	A	L2	0	-2	-2	4	4
2	M	76	C5	B	C	C7	23	22	55	-1	-2
3	M	46	C7	A	D	C7	20	26	46	44	44
7	M	79	C4	C	D	C4	39	11	50	0	0
8	F	45	C4	A	A	C4	10	10	20	0	0
9	M	25	T11	A	A	T11	0	0	0	0	0
10	M	60	C5	A	B	C4	-5	-1	-6	-1	-1
11	M	22	C5	A	A	C5	0	0	0	0	0
12	F	69	C5	C	D	C5	0	16	16	0	0
13	M	22	T6	A	A	T6	0	0	0	0	0
14	M	68	C5	B	C	C4	6	11	17	0	0
15	M	35	C6	B	D	C6	3	32	35	0	0
17	M	59	C3	A	A	C3	2	0	2	2	2
19	M	47	C4	A	B	C4	6	0	6	2	3
20	M	34	T4	A	A	T4	0	0	0	0	0
21	F	32	C3	A	A	C2	0	0	0	-4	-4
22	M	52	C4	B	C	C4	18	18	36	20	60
24	M	49	C7	B	C	C4	-17	20	3	33	32
27	M	68	C4	C	D	C8	48	4	52	50	50
28	M	59	C4	C	A	C4	0	5	5	22	22
29	M	78	C4	C	D	C4	28	12	40	0	0
31	M	57	C4	B	D	C4	37	28	65	48	48
33	M	28	C5	C	D	C5	2	30	32	9	0
34	M	30	C4	B	C	C4	3	25	28	8	8
35	M	71	C4	A	B	C4	0	0	0	14	14

Supplemental Table **2 The hub genes identified by WGCNA.** The hub genes were filtered by |MM| > 0.8 and |GS| > 0.2.

Model	Number	hub genes
purple	6	hsa-miR-6746-3p, hsa-miR-451a, hsa-miR-197-3p, hsa-miR-424-3p, hsa-miR-877-3p, hsa-miR-7111-3p
midnightblue	7	hsa-miR-9-3p, hsa-miR-136-3p, hsa-miR-1185-1-3p, hsa-miR-374b-5p, hsa-miR-4290, hsa-miR-374c-3p, hsa-miR-6809-3p
cyan	7	hsa-miR-125b-1-3p, hsa-miR-485-5p, hsa-miR-1306-5p, hsa-miR-3154, hsa-miR-5009-5p, hsa-miR-5193, hsa-miR-6741-3p
turquoise	29	hsa-miR-99a-5p, hsa-miR-29a-3p, hsa-miR-103a-3p, hsa-miR-103b, hsa-miR-29b-3p, hsa-miR-449b-5p, hsa-miR-210-3p, hsa-miR-125a-5p, hsa-let-7d-5p, hsa-miR-141-3p, hsa-miR-200c-3p, hsa-miR-15b-5p, hsa-miR-449a, hsa-miR-10b-5p, hsa-miR-21-5p, hsa-miR-429, hsa-miR-449c-5p, hsa-miR-31-5p, hsa-miR-24-3p, hsa-miR-34c-5p, hsa-miR-7-5p, hsa-miR-3529-3p, hsa-miR-20a-5p, hsa-miR-361-5p, hsa-miR-191-5p, hsa-miR-200b-3p, hsa-miR-130a-3p, hsa-miR-99b-5p, hsa-miR-3182
Brown	23	hsa-miR-4787-5p, hsa-miR-7853-5p, hsa-miR-32-5p, hsa-miR-200a-5p, hsa-miR-6869-5p, hsa-miR-6126, hsa-miR-302a-3p, hsa-miR-302b-3p, hsa-miR-28-5p, hsa-miR-342-3p, hsa-miR-365a-3p, hsa-miR-365b-3p, hsa-miR-4785, hsa-miR-324-5p, hsa-miR-1303, hsa-miR-8485, hsa-miR-27a-5p, hsa-miR-32-3p, hsa-miR-5196-5p, hsa-miR-4429, hsa-miR-941, hsa-miR-518b, hsa-miR-148b-3p
blue	14	hsa-miR-133a-3p, hsa-miR-133b, hsa-let-7i-5p, hsa-miR-1-3p, hsa-miR-378a-3p, hsa-miR-22-5p, hsa-miR-6529-5p, hsa-miR-199a-5p, hsa-miR-193b-3p, hsa-miR-30e-3p, hsa-miR-378c, hsa-miR-133a-5p, hsa-miR-206, hsa-miR-27b-3p

Supplemental Table **3 miRNA mimic/inhibitor sequences**

Cat. Number	Description microRNA mimics	Estimated bus sequence
C-300491-03	Human hsa- miR-20a-5p	UAAAGUGCUUAUAGUGCAGGUAG
C-300723-03	Human hsa- miR-449a	UGGCAGUGUAUUGUUAGCUGGU
C-301180-01	Human hsa- miR-16-2-3p	CCAAUAUUACUGUGCUGCUUUA
C-300682-05	Human hsa- miR-375	UUUGUUCGUUCGGCUCGCGUGA
C-300542-05	Human hsa- miR-30c-5p	UGUAAACAUCCUACACUCUCAGC
C-300664-05	Human hsa- miR-362-5p	AAUCCUUGGAACCUAGGUGUGAGU
C-303064-00	Human hsa- miR-6873-3p	UUCUCUCUGUCUUUCUCUCUCAG
C-301238-01	Human hsa- miR-708-5p	AAGGAGCUUACAAUCUAGCUGGG
IH-303098-01	Human has- miR-7111-3p inhibitor	AUCCUCUCUUCCCUCCUCCCAG
IH-300643-05	Human hsa- miR-195-5p inhibitor	UAGCAGCACAGAAAUAUUGGC
CN-001000-01	miRIDIAN microRNA Mimic Negative Control #1	

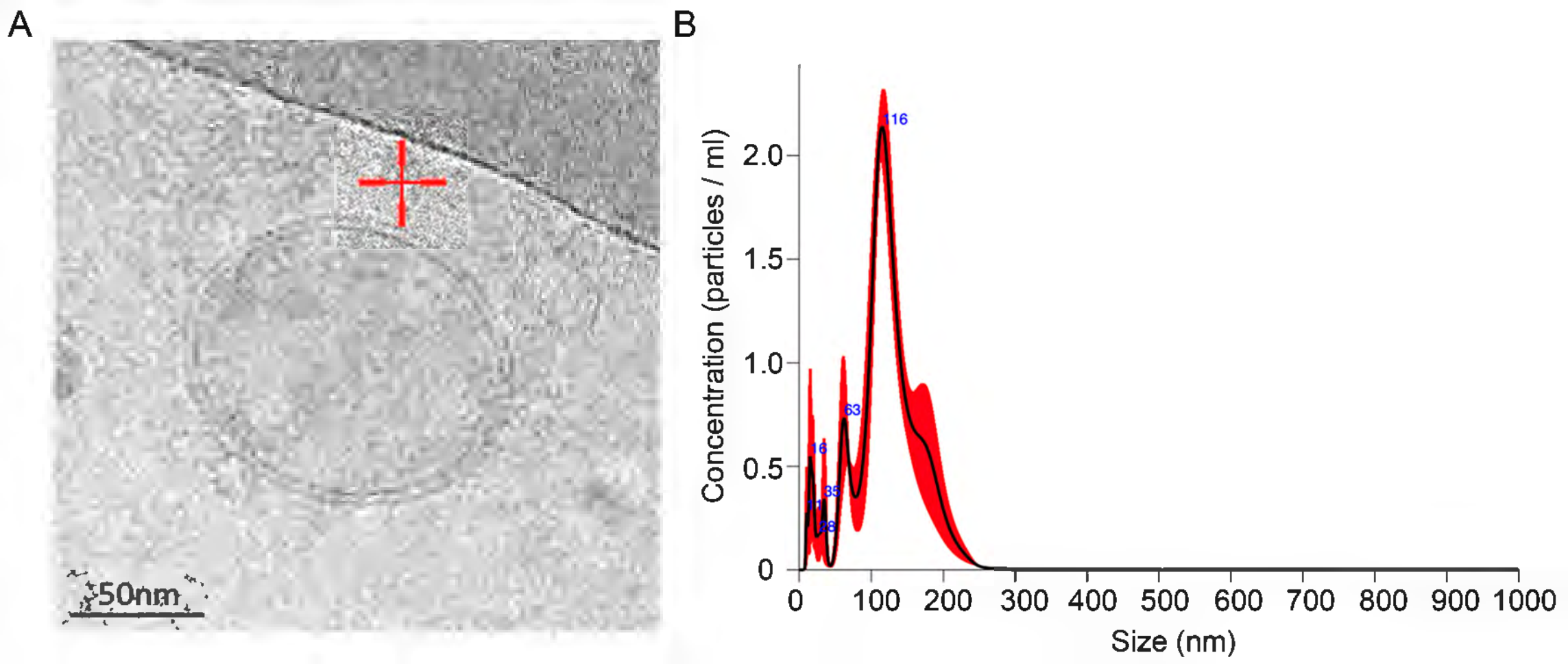

A
50nm
B
Concentration (particles / ml)
Size (nm)
116
63
16
35
11
28
0
0.5
1.0
1.5
2.0
0
100
200
300
400
500
600
700
800
900
1000

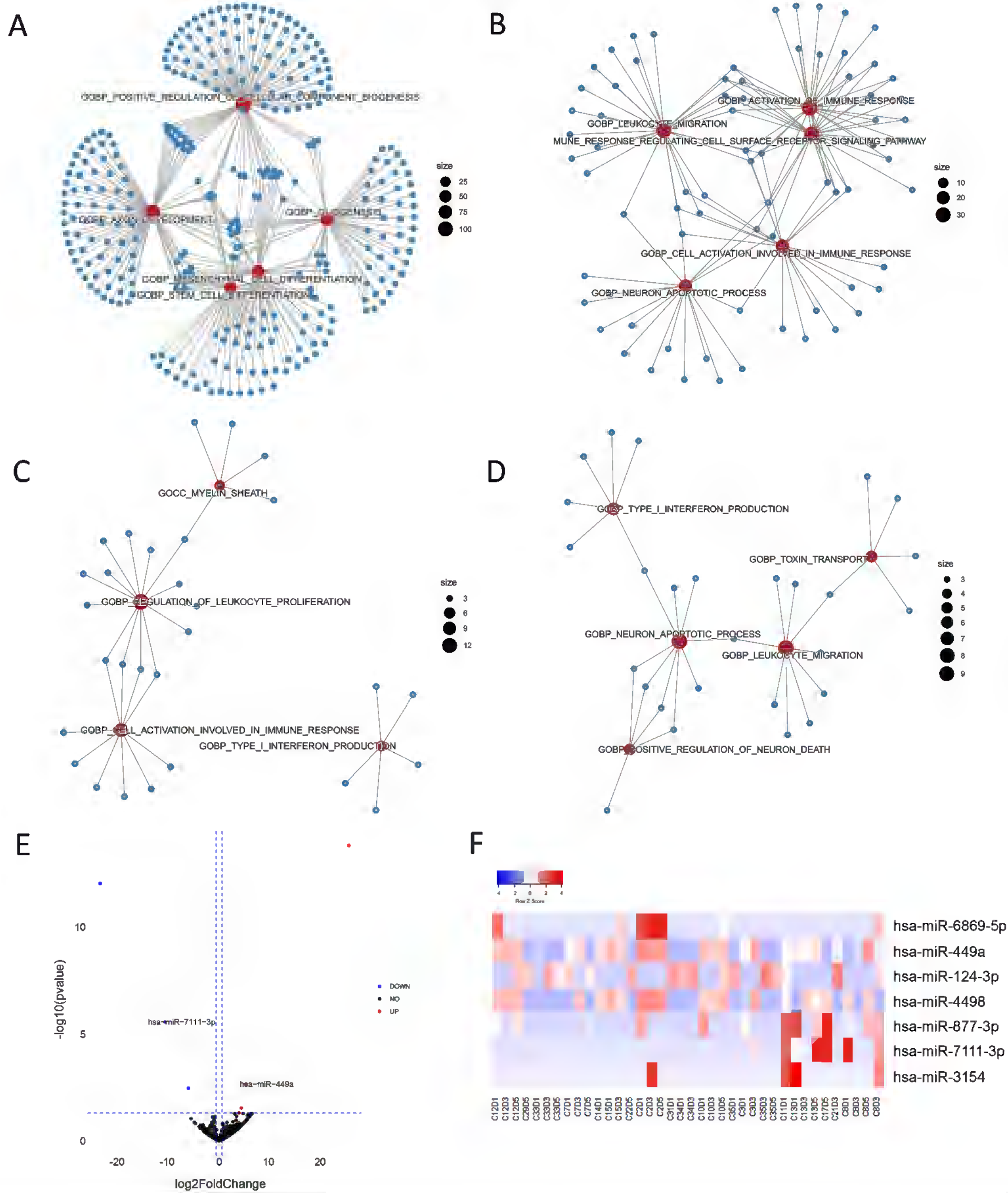

A
size
25
50
75
100
B
GOBP_ACTIVATION_OF_IMMUNE_RESPONSE
GOBP_LEUKOCYTE_MIGRATION
MUNE_RESPONSE_REGULATING_CELL_SURFACE_RECEPTOR_SIGNALING_PATHWAY
GOBP_CELL_ACTIVATION_INVOLVED_IN_IMMUNE_RESPONSE
GOBP_NEURON_APOPTOTIC_PROCESS
size
10
20
30
C
GOCC_MYELIN_SHEATH
GOBP_REGULATION_OF_LEUKOCYTE_PROLIFERATION
GOBP_CELL_ACTIVATION_INVOLVED_IN_IMMUNE_RESPONSE
GOBP_TYPE_I_INTERFERON_PRODUCTION
size
3
6
9
12
D
GOBP_TYPE_I_INTERFERON_PRODUCTION
GOBP_TOXIN_TRANSPORT
GOBP_NEURON_APOPTOTIC_PROCESS
GOBP_LEUKOCYTE_MIGRATION
GOBP_POSITIVE_REGULATION_OF_NEURON_DEATH
size
3
4
5
6
7
8
9
E
-log10(pvalue)
log2FoldChange
hsa-miR-7111-3p
hsa-miR-449a
DOWN
NO
UP
F
hsa-miR-6869-5p
hsa-miR-449a
hsa-miR-124-3p
hsa-miR-4498
hsa-miR-877-3p
hsa-miR-7111-3p
hsa-miR-3154

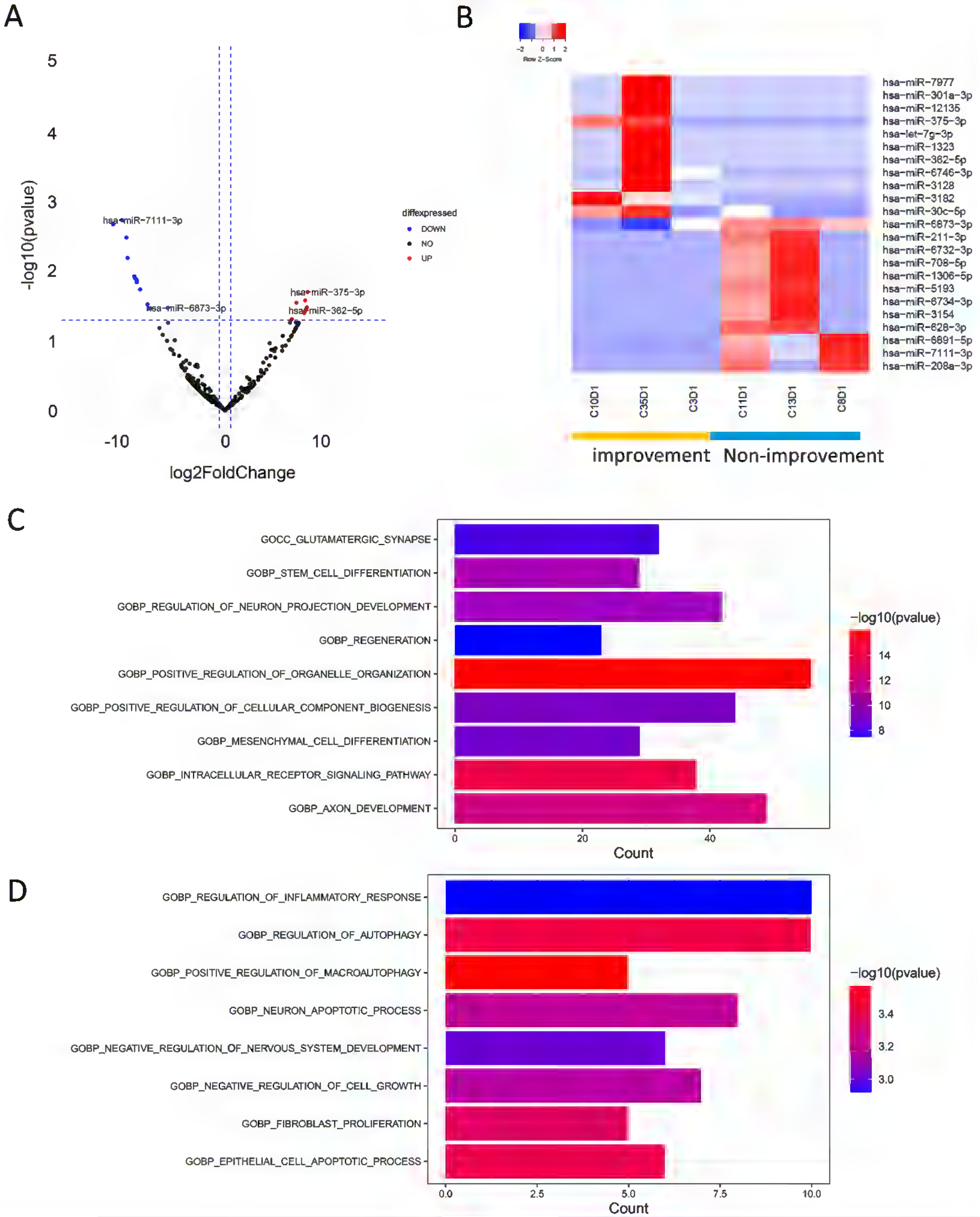

A
B
C
D
-log10(pvalue)
log2FoldChange
hsa-miR-7111-3p
hsa-miR-6873-3p
hsa-miR-375-3p
hsa-miR-362-5p
diffexpressed
DOWN
NO
UP
Row Z-Score
hsa-miR-7977
hsa-miR-301a-3p
hsa-miR-12135
hsa-miR-375-3p
hsa-let-7g-3p
hsa-miR-1323
hsa-miR-362-5p
hsa-miR-6746-3p
hsa-miR-3128
hsa-miR-3182
hsa-miR-30c-5p
hsa-miR-6873-3p
hsa-miR-211-3p
hsa-miR-6732-3p
hsa-miR-708-5p
hsa-miR-1306-5p
hsa-miR-5193
hsa-miR-6734-3p
hsa-miR-3154
hsa-miR-628-3p
hsa-miR-6891-5p
hsa-miR-7111-3p
hsa-miR-208a-3p
C10D1
C35D1
C3D1
C11D1
C13D1
C8D1
improvement
Non-improvement
GOCC_GLUTAMATERGIC_SYNAPSE
GOBP_STEM_CELL_DIFFERENTIATION
GOBP_REGULATION_OF_NEURON_PROJECTION_DEVELOPMENT
GOBP_REGENERATION
GOBP_POSITIVE_REGULATION_OF_ORGANELLE_ORGANIZATION
GOBP_POSITIVE_REGULATION_OF_CELLULAR_COMPONENT_BIOGENESIS
GOBP_MESENCHYMAL_CELL_DIFFERENTIATION
GOBP_INTRACELLULAR_RECEPTOR_SIGNALING_PATHWAY
GOBP_AXON_DEVELOPMENT
Count
-log10(pvalue)
GOBP_REGULATION_OF_INFLAMMATORY_RESPONSE
GOBP_REGULATION_OF_AUTOPHAGY
GOBP_POSITIVE_REGULATION_OF_MACROAUTOPHAGY
GOBP_NEURON_APOPTOTIC_PROCESS
GOBP_NEGATIVE_REGULATION_OF_NERVOUS_SYSTEM_DEVELOPMENT
GOBP_NEGATIVE_REGULATION_OF_CELL_GROWTH
GOBP_FIBROBLAST_PROLIFERATION
GOBP_EPITHELIAL_CELL_APOPTOTIC_PROCESS
Count
-log10(pvalue)

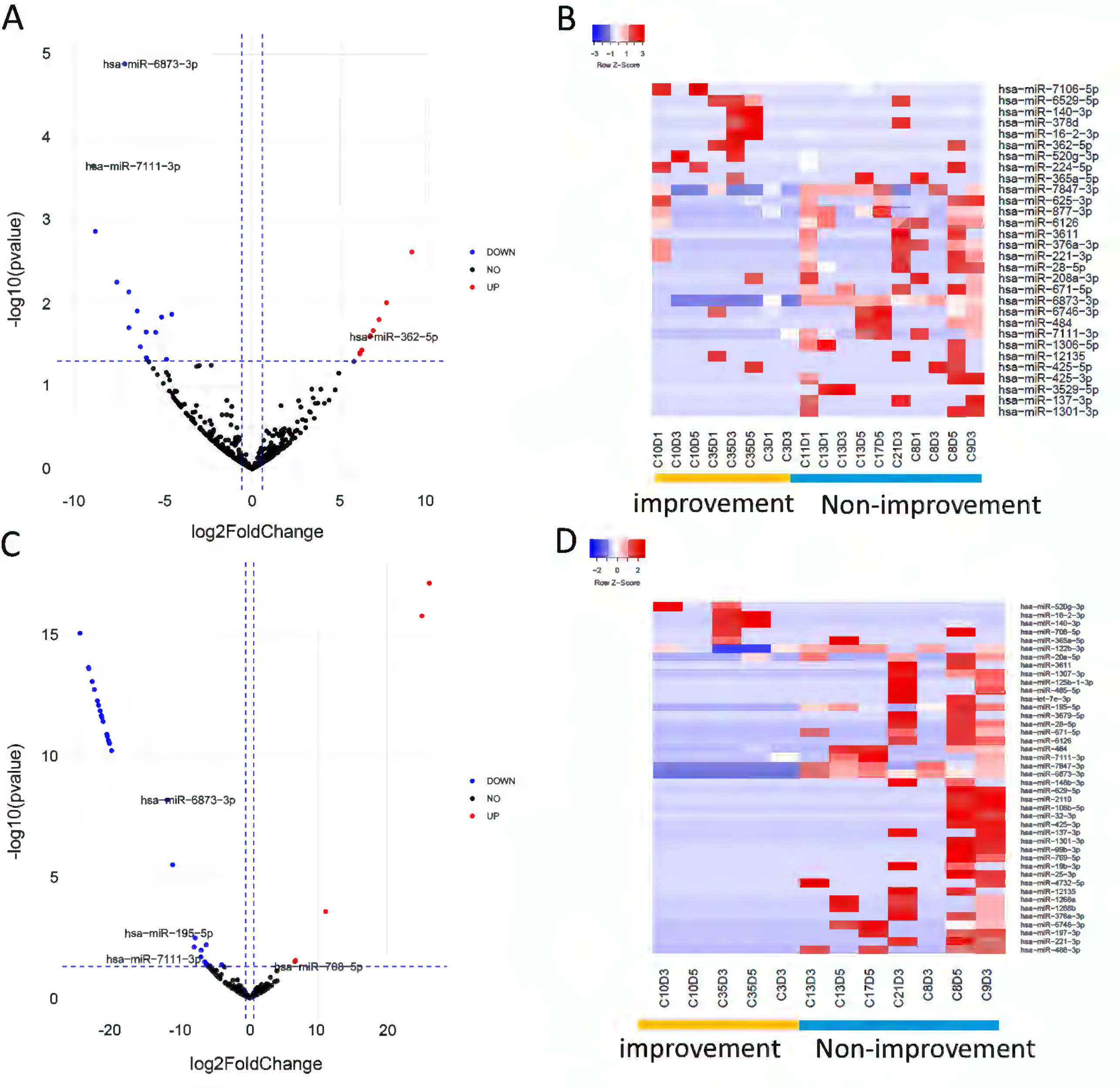

A
B
C
D
5
4
3
2
1
0
-log10(pvalue)
-10
-5
0
5
10
log2FoldChange
hsa-miR-6873-3p
hsa-miR-7111-3p
hsa-miR-362-5p
DOWN
NO
UP
-3 -1 1 3
Row Z-Score
hsa-miR-7106-5p
hsa-miR-6529-5p
hsa-miR-140-3p
hsa-miR-378d
hsa-miR-16-2-3p
hsa-miR-362-5p
hsa-miR-520g-3p
hsa-miR-224-5p
hsa-miR-365a-5p
hsa-miR-7847-3p
hsa-miR-625-3p
hsa-miR-877-3p
hsa-miR-6126
hsa-miR-3611
hsa-miR-376a-3p
hsa-miR-221-3p
hsa-miR-28-5p
hsa-miR-208a-3p
hsa-miR-671-5p
hsa-miR-6873-3p
hsa-miR-6746-3p
hsa-miR-484
hsa-miR-7111-3p
hsa-miR-1306-5p
hsa-miR-12135
hsa-miR-425-5p
hsa-miR-425-3p
hsa-miR-3529-5p
hsa-miR-137-3p
hsa-miR-1301-3p
C10D1
C10D3
C10D5
C35D1
C35D3
C35D5
C3D1
C3D3
C11D1
C13D1
C13D3
C13D5
C17D5
C21D3
C8D1
C8D3
C8D5
C9D3
improvement
Non-improvement
15
10
5
0
-log10(pvalue)
-20
-10
0
10
20
log2FoldChange
hsa-miR-6873-3p
hsa-miR-195-5p
hsa-miR-7111-3p
hsa-miR-708-5p
DOWN
NO
UP
-2 0 2
Row Z-Score
hsa-miR-520g-3p
hsa-miR-16-2-3p
hsa-miR-140-3p
hsa-miR-708-5p
hsa-miR-365a-5p
hsa-miR-122b-3p
hsa-miR-20a-5p
hsa-miR-3611
hsa-miR-1307-3p
hsa-miR-125b-1-3p
hsa-miR-465-5p
hsa-let-7e-3p
hsa-miR-195-5p
hsa-miR-3679-5p
hsa-miR-28-5p
hsa-miR-671-5p
hsa-miR-6126
hsa-miR-484
hsa-miR-7111-3p
hsa-miR-7847-3p
hsa-miR-6873-3p
hsa-miR-148b-3p
hsa-miR-629-5p
hsa-miR-2110
hsa-miR-106b-5p
hsa-miR-32-3p
hsa-miR-425-3p
hsa-miR-137-3p
hsa-miR-1301-3p
hsa-miR-99b-3p
hsa-miR-769-5p
hsa-miR-19b-3p
hsa-miR-25-3p
hsa-miR-4732-5p
hsa-miR-12135
hsa-miR-1268a
hsa-miR-1268b
hsa-miR-376a-3p
hsa-miR-6746-3p
hsa-miR-197-3p
hsa-miR-221-3p
hsa-miR-488-3p
C10D3
C10D5
C35D3
C35D5
C3D3
C13D3
C13D5
C17D5
C21D3
C8D3
C6D5
C9D3
improvement
Non-improvement

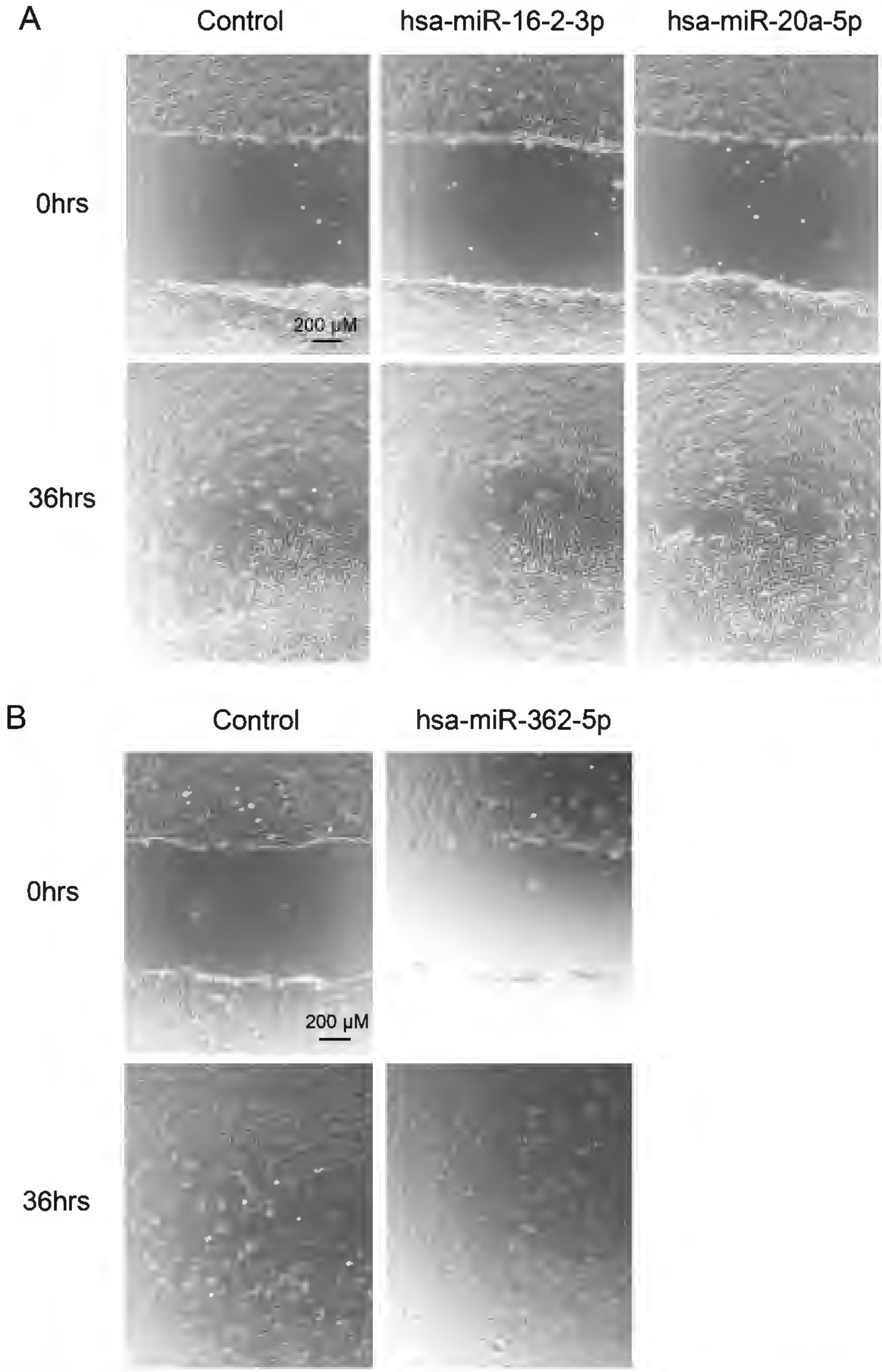
A
Control
hsa-miR-16-2-3p
hsa-miR-20a-5p
0hrs
200 μM
36hrs
B
Control
hsa-miR-362-5p
0hrs
200 μM
36hrs

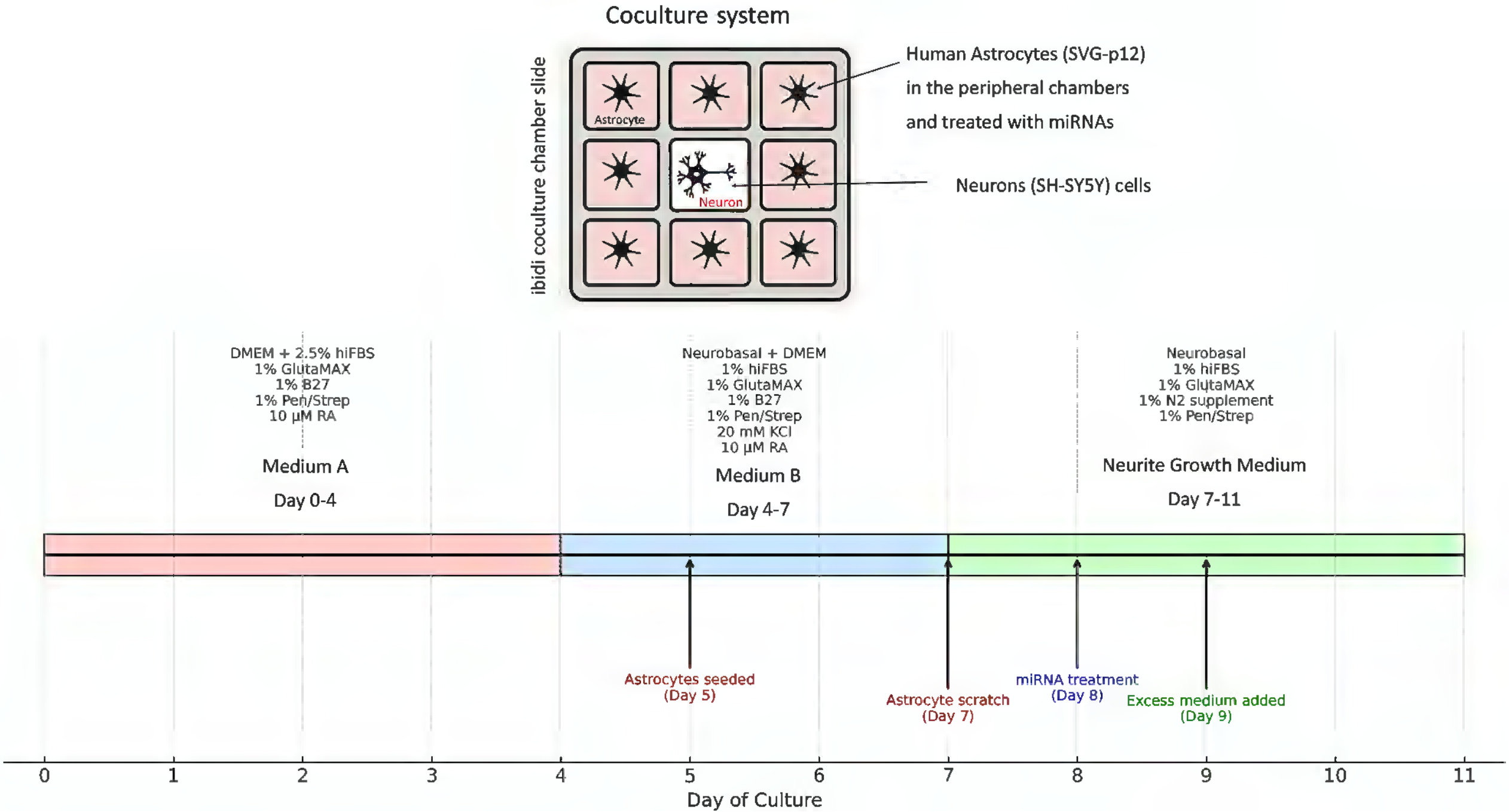
Coculture system
ibidi coculture chamber slide
Astrocyte
Neuron
Human Astrocytes (SVG-p12)
in the peripheral chambers
and treated with miRNAs
Neurons (SH-SY5Y) cells
DMEM + 2.5% hiFBS
1% GlutaMAX
1% B27
1% Pen/Strep
10 μM RA
Medium A
Day 0-4
Neurobasal + DMEM
1% hiFBS
1% GlutaMAX
1% B27
1% Pen/Strep
20 mM KCl
10 μM RA
Medium B
Day 4-7
Neurobasal
1% hiFBS
1% GlutaMAX
1% N2 supplement
1% Pen/Strep
Neurite Growth Medium
Day 7-11
Astrocytes seeded
(Day 5)
Astrocyte scratch
(Day 7)
miRNA treatment
(Day 8)
Excess medium added
(Day 9)
0
1
2
3
4
5
6
7
8
9
10
11
Day of Culture

Supplemental Figure 1 (A) Cryo-EM images of EVs isolated from human CSF of spinal cord injury patients. Scale bar: 50 nm. (B) Nano-tracking analysis (NTA) of CSF.

Supplemental Figure 2 (A-D) The network in each module visualization highlights enriched functional categories and their associated genes. Functional enrichment analysis (GO) for module Blue (A), Cyan (B), Midnight blue (C) and Purple (D) was performed based on the target genes of hub miRNAs. Target genes of these hub miRNAs were determined using multiMiR, referencing TarBase (v9.0) and miRTarBase (v9.0). Hub miRNAs were identified in the module using the criteria $|MM| > 0.8$ and $|GS| > 0.2$. Blue dots indicate DEGs with fold-changes ≤ -1.5 and p-value < 0.05, while red dots represent DEGs with fold-changes ≥ 1.5 and p-value < 0.05 (E) Volcano plot and (F) heatmap of differentially expressed genes (DEGs) between patients with improvement and non-improvement injuries among all CSF samples.

Supplemental Figure 3 (A) Volcano plot and (B) heatmap of DEGs between patients with improvement and non-improvement injuries on day 1. Blue dots indicate DEGs with fold-changes ≤ -1.5 and p-value < 0.05, while red dots represent DEGs with fold-changes ≥ 1.5 and p-value < 0.05. (C, D) Gene ontology enrichment analysis of significant miRNA target genes, highlighting enrichment in the improvement group (C) and the non-improvement group (D). Bar plot color represents the -log10(p-value), while "Count" indicates the number of genes enriched in each GO term.

Supplemental Figure 4 (A-D). The Volcano plot and heatmap of DEGs between patients with improvement and non-improvement injuries on day 1/3/5 with samples from day1, day 3 and day 5 combined (A, B) and day 3/5, with samples from day 3 and day 5 combined (C, D)

Supplemental Figure 5 Results of scratch assay at 36 hours without significant changes after miRNA mimic or inhibitor treatments. A and B: images of scratch area from groups at 0- and 36-hour time points

Supplemental Figure 6 Diagram illustrating the workflow of astrocyte-neuron co-culture
